# Synthesis and Mechanistic Studies of a Novel Homoisoflavanone Inhibitor of Endothelial Cell Growth

**DOI:** 10.1371/journal.pone.0095694

**Published:** 2014-04-21

**Authors:** Halesha D. Basavarajappa, Bit Lee, Xiang Fei, Daesung Lim, Breedge Callaghan, Julie A. Mund, Jamie Case, Gangaraju Rajashekhar, Seung-Yong Seo, Timothy W. Corson

**Affiliations:** 1 Eugene and Marilyn Glick Eye Institute, Department of Ophthalmology, Indiana University School of Medicine, Indianapolis, Indiana, United States of America; 2 Department of Biochemistry and Molecular Biology, Indiana University School of Medicine, Indianapolis, Indiana, United States of America; 3 College of Pharmacy, Gachon University, Incheon, South Korea; 4 Department of Pediatrics, Indiana University School of Medicine, Indianapolis, Indiana, United States of America; 5 Indiana University Melvin and Bren Simon Cancer Center, Indianapolis, Indiana, United States Of America; 6 Department of Cellular and Integrative Physiology, Indiana University School of Medicine, Indianapolis, Indiana, United States of America; 7 Department of Pharmacology and Toxicology, Indiana University School of Medicine, Indianapolis, Indiana, United States of America; Purdue University, United States of America

## Abstract

Preventing pathological ocular angiogenesis is key to treating retinopathy of prematurity, diabetic retinopathy and age-related macular degeneration. At present there is no small molecule drug on the market to target this process and hence there is a pressing need for developing novel small molecules that can replace or complement the present surgical and biologic therapies for these neovascular eye diseases. Previously, an antiangiogenic homoisoflavanone was isolated from the bulb of a medicinal orchid, *Cremastra appendiculata*. In this study, we present the synthesis of a novel homoisoflavanone isomer of this compound. Our compound, SH-11052, has antiproliferative activity against human umbilical vein endothelial cells, and also against more ocular disease-relevant human retinal microvascular endothelial cells (HRECs). Tube formation and cell cycle progression of HRECs were inhibited by SH-11052, but the compound did not induce apoptosis at effective concentrations. SH-11052 also decreased TNF-α induced p38 MAPK phosphorylation in these cells. Intriguingly, SH-11052 blocked TNF-α induced IκB-α degradation, and therefore decreased NF-κB nuclear translocation. It decreased the expression of NF-κB target genes and the pro-angiogenic or pro-inflammatory markers VCAM-1, *CCL2*, *IL8*, and *PTGS2*. In addition SH-11052 inhibited VEGF induced activation of Akt but not VEGF receptor autophosphorylation. Based on these results we propose that SH-11052 inhibits inflammation induced angiogenesis by blocking both TNF-α and VEGF mediated pathways, two major pathways involved in pathological angiogenesis. Synthesis of this novel homoisoflavanone opens the door to structure-activity relationship studies of this class of compound and further evaluation of its mechanism and potential to complement existing antiangiogenic drugs.

## Introduction

Angiogenesis is a tightly controlled, complex physiological process involving the formation of new blood vessels from pre-existing ones. Under normal conditions, angiogenesis does not occur in the body, except during development and wound repair processes. However, during numerous pathological conditions angiogenesis occurs, notably in ocular diseases such as retinopathy of prematurity (ROP), diabetic retinopathy (DR), and “wet” age-related macular degeneration (AMD). Collectively, these diseases are a major cause of blindness through the lifespan [1].

Clinical symptoms of DR are seen in 75% of diabetic patients, with 10% of them eventually developing visual impairment [1]. DR is currently the leading cause of blindness among working age adults and accounts for 8% of the legal blindness in the United States [1]. Similarly, AMD is responsible for causing blindness in elderly people worldwide. Wet AMD affects approximately 2 million people in the United States and is estimated to engender a productivity burden of nearly $5.4 billion [2]. In the United States, 70% of the infants with very low birth weight develop ROP after exposure to postnatal hyperoxia. Around 1,300 infants per year in the United States develop complete vision loss due to this disease, and 500 more are severely impaired [3]. Overall, between 6% and 18% of childhood blindness is attributable to ROP [4]. Moreover, as more and more children survive premature birth in middle income countries due to improvements in neonatal intensive care, ROP is becoming more prevalent worldwide [4]. Thus, these three diseases, along with other neovascular diseases of the eye, represent a significant public health problem.

After pathological angiogenesis, newly formed blood vessels are fragile, porous and not fully differentiated. The formation of such new blood vessels in the eye leads to various complications such as hemorrhage, retinal detachment, fibrotic scarring and rapid photoreceptor degradation. If these conditions are left untreated, irreversible vision loss can occur. Cryotherapy, laser therapy and surgical interventions are major treatment options for neovascular eye diseases, yet they are also associated with vision-damaging side effects [5]. Recently, drug therapy using biologics such as bevacizumab, pegaptanib and aflibercept has been successful in treating these diseases [5], [6]. An antibody, aptamer, and fusion protein respectively, these drugs all act by inhibiting vascular endothelial growth factor (VEGF) signaling, a key pro-angiogenic signaling pathway [1]. However these medications face an unfavorable cost to benefit ratio [7], [8] and have the potential for significant acute systemic side effects such as non-ocular hemorrhage and myocardial infarction [5]. There is also a significant population that is refractory to these drugs; up to 45% in one series of AMD patients [9]. In addition, during pathological conditions, levels of inflammatory cytokines such as TNF-α and IL-1 are elevated and these cytokines in turn promote angiogenesis along with VEGF [10], [11]. Hence targeting not only VEGF signaling but multiple proangiogenic signals is required to improve the efficacy of treatment for diseases arising from pathological angiogenesis. At present there is currently no small molecule drug on the market to specifically prevent angiogenesis in the eye, hence there is a pressing need to develop specific novel small molecule drugs to treat these blinding eye diseases.

**Figure 1:**
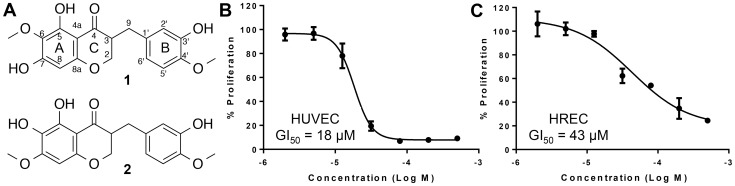
Homoisoflavanone SH-11052 inhibits proliferation of endothelial cells. The effects of SH-11052 (**2**), an isomer of the natural-source homoisoflavanone **1** (A) on the proliferation of HUVECs (B) and HRECs (C) were tested by alamarBlue fluorescence in the concentration range of 0.5 nM to 500 µM and shown as mean ± SEM relative to DMSO control. Dose response curves and indicated GI_50_ values were generated using GraphPad Prism software.

Several small molecules, including natural products, have been identified that inhibit pathological angiogenesis such as artemisinin, curcumin, fumagillin, LLL12, panduratin, decursin, withaferin, and sunitinib [12]–[18]. A recent addition to this group is a homoisoflavanone, 5,7-dihydroxy-3-(3-hydroxy-4-methoxybenzyl)-6-methoxychroman-4-one that was isolated from the plant *Cremastra appendiculata* (D. Don) (Compound **1**; Fig. 1A). The bulb of the orchid *C. appendiculata* is a traditional medicine in East Asia, used internally to treat several cancers, and externally for skin lesions [19]–[21]. This compound has also been isolated from members of the Hyacinthaceae, a rich source of homoisoflavanones, which are a small class of naturally occurring heterocyclic compounds that are structurally similar to isoflavonoids [22].

Compound **1** was shown to possess anti-angiogenic activity both *in vitro* and *in vivo* [21], [23], [24]. Shim et al. identified compound **1** as a potent inhibitor of the proliferation of human umbilical vein endothelial cells (HUVECs) [21]. Later it was shown that compound **1** inhibited both vascular tube formation and migration of HUVECs induced by basic fibroblast growth factor (bFGF) *in vitro*. In the chick chorioallantoic membrane model, compound **1** was as effective as retinoic acid in blocking new vessel growth induced by bFGF [21]. The anti-angiogenic property of the compound as isolated from the plant extract was further confirmed *in vivo* in the laser-induced choroidal neovascularization and oxygen induced retinopathy mouse models, used for treatment evaluations in neovascular AMD and in ROP, respectively [23], [24]. Importantly, injection of compound **1** into the vitreous of normal adult mice showed no short-term cytotoxic or inflammatory effects on the retina, nor did it induce apoptosis of retinal cells [23]. These results suggest that proliferative ocular vascular diseases such as ROP, DR, and AMD may be targeted using compound **1** or its derivatives.

In order to further explore the potential of homoisoflavanones as treatments for neovascular eye diseases, we synthesized a novel isomer of compound **1**, 5,6-dihydroxy-3-(3-hydroxy-4-methoxybenzyl)-7-methoxychroman-4-one, known as SH-11052 (compound **2**, Fig. 1A). In the present study we report this synthesis and show the anti-angiogenic properties of compound **2** in human retinal microvascular endothelial cells (HRECs). We also demonstrate that compound **2** blocks TNF-α induced NF-κB signaling and the VEGF-induced PI3K/Akt pathway, two major proangiogenic signaling pathways activated during inflammation induced angiogenesis. These results suggest that the compound exerts its anti-angiogenic properties by blocking inflammation-induced angiogenic pathways.

## Materials and Methods

### Materials Used

HRECs and Attachment Factor were purchased from Cell Systems (Kirkland, WA, USA). Clonetics HUVECs were purchased from Lonza (Walkersville, MD, USA). All cells were used between passages 5 and 7. Endothelial Growth Medium (EGM-2) was prepared by mixing the contents of an EGM-2 “Bullet Kit” (Cat no. CC-4176) with Endothelial Basal Medium (EBM) (Lonza). The EGM-2 “Bullet Kit” contains hydrocortisone, human fibroblast growth factor (hFGF), VEGF, R3-insulin like growth factor (R3-IGF-1), ascorbic acid, human epidermal growth factor (hEGF), gentamycin and heparin along with 2% fetal bovine serum (FBS). Compound BAY 11-7082, caffeic acid phenethyl ester (CAPE), TNF-α, and α-tubulin antibody were from Sigma (St. Louis, MO, USA), and human VEGF-165 was from BioLegend (San Diego, CA, USA). The antibodies for p38 MAPK, NF-κB p65 and VCAM-1 were obtained from Santa Cruz (Dallas, TX, USA) while the cleaved caspase-3, phospho p38 MAPK, Akt, phospho-Akt, VEGFR2, phospho-VEGFR2, and IκB-α antibodies were from Cell Signaling (Danvers, MA, USA). Secondary antibodies were from Thermo Fisher Scientific (Pittsburgh, PA, USA). The TaqMan probes and 5′-ethynyl-2′-deoxyuridine (EdU) incorporation assay kit were procured from Life Technologies (Carlsbad, CA, USA). AbD Serotec (Kidlington, UK) was the source of the alamarBlue, while BD Biosciences (San Jose, CA, USA) supplied the Matrigel. The Bradford reagent for protein estimation was prepared by dissolving 0.3 g of Coomassie G-250 (Pierce) in 500 mL of 3% perchloric acid.

### Synthesis of SH-11052

Full details of synthetic methodology and chemical characterization of all intermediates and the final compound **2** is provided in Methods S1 in File S1. Assignment was confirmed by 2D-NMR (Figure S1 in File S1).

### Cell Proliferation Assay

In a 96-well clear bottom black plate, cells (2,500 cells per well) were seeded in a total volume of 100 µL EGM-2. After 24 hours of incubation of the plate at 37°C and 5% CO_2_, a DMSO solution of compound **2**, BAY 11-7082, or CAPE was added in the concentration range of 0.5 nM to 500 µM (final DMSO concentration = 1%). The plate was then further incubated for 48 hours before adding 11.1 µL of alamarBlue reagent to each well. Four hours after the addition of alamarBlue, fluorescence readings with excitation and emission wavelengths of 560 nm and 590 nm respectively were taken and the data were analyzed in GraphPad Prism software (v. 6.0). Dose response curves were generated and the GI_50_ values were calculated using the following equation: *Y* = 100/(1+10∧(*X*−Log(GI_50_))).

### EdU Incorporation Assay

Cells (25,000 per coverslip) were seeded onto coverslips coated with Attachment Factor placed in a 6-well plate and incubated with the indicated concentrations of compound **2** in EGM-2 for 24 hours at 37°C and 5% CO_2_. The cells were then serum starved for 8 hours and the medium was replaced with EGM-2 containing 10 µM EdU. The plate was further incubated for another 8 hours before processing the cells for detection of labeled DNA (according to the manufacturer's instructions for the Click-iT EdU assay kit). Images were taken using an EVOS fluorescence microscope (AMG, Mill Creek, WA, USA) and the number of DAPI stained and EdU stained cells were counted in six randomly chosen fields using ImageJ software.

### *In Vitro* Angiogenesis Assay

Matrigel assays were performed as previously described [25], with slight modifications for the use of HRECs. Briefly, HRECs were starved overnight at 0.5% FBS in EBM-2 and plated on a 96-well plate at a density of 7,500 cells/well over 50 µL of Matrigel high concentration basement membrane. Compound **2** was added at the indicated concentrations in EBM-2+1% FBS. Cells were observed every 2 hours by bright field microscopy at 40× magnification for tube formation. Closed units (polygons) were manually counted at 8 hours post plating and numbers normalized to the DMSO control. Assays were performed in triplicate.

### Caspase-3 Immunofluorescence Assay

Cells (25,000 per coverslip) were seeded onto coverslips coated with Attachment Factor and incubated at 37°C and 5% CO_2_ in EGM-2 until ∼80% confluence was achieved. The cells were then incubated for 4 hours with the indicated concentrations of compound **2**. Staurosporine (SP; 1 µM) was used as a positive control. After the incubation, the cells were fixed in 4% paraformaldehyde for 20 min at room temperature followed by three quick washes in Tris buffered saline pH 7.4 (TBS). The cells were permeabilized by incubating with 0.5% Triton X-100 for 10 minutes and then blocked in 10% block solution (DAKO, Glostrup, Denmark) in TBS plus 1% bovine serum albumin (BSA) for 1 hour. The cells were then incubated with cleaved caspase-3 (D175) antibody (1∶200 dilution) overnight at 4°C. Dylight 555 conjugated goat anti-rabbit secondary antibody (1∶400) was used to probe the cleaved caspase-3 antibody. The coverslips were mounted using Vectashield mounting medium containing DAPI (Vector Labs, Burlingame, CA, USA) for nuclear staining. The cells were imaged using an LSM 700 confocal microscope (Zeiss, Thornwood, NY, USA).

### NF-κB Nuclear Translocation Assay

Cells (25,000 per coverslip) were seeded onto coverslips coated with Attachment Factor and incubated at 37°C and 5% CO_2_ for 24 hours in EGM-2. The cells were starved in 0.1% serum-EBM-2 for 8 hours followed by 0.1% serum-EBM-2 medium for one hour in the presence of different concentrations of compound **2**. The cells were induced with 10 ng/ml TNF-α for 20 minutes and fixed with 4% paraformaldehyde solution for 20 min at room temperature. Cells were quickly washed three times in TBS and permeabilized by incubating with 0.5% Triton X-100 for 10 minutes. The cells were blocked in 10% block solution (DAKO) in TBS plus 1% BSA followed by incubation with an antibody against NF-κB p65 (1∶50 dilution). Dylight 488-conjugated goat anti-mouse secondary antibody (1∶200 dilution) was used to probe the NF-κB p65 antibody. The coverslips were mounted using Vectashield mounting medium containing DAPI (Vector Labs) for nuclear staining. The cells were imaged using an LSM 700 confocal microscope (Zeiss).

### VCAM-1 Expression Study

Cells (25,000 per coverslip) were seeded onto coverslips coated with Attachment Factor and incubated at 37°C and 5% CO_2_ for 24 hours in EGM-2. The cells were starved in 0.1% serum-EBM-2 for 8 hours followed by incubation in 0.1% serum-EBM-2 medium for an hour in the presence of different concentrations of compound **2**. The cells were challenged with 10 ng/ml of TNF-α for 24 hours and fixed with 4% paraformaldehyde solution for 20 minutes at room temperature. The coverslips were quickly washed three times in TBS and blocked using 10% block solution (DAKO) prepared in 1× TBS-1% BSA buffer. The coverslips were incubated with the antibody against VCAM-1 (1∶100 dilution) for 16 hours at 4°C followed by three washes in TBS- 0.1% BSA buffer. Dylight 555-conjugated secondary antibody (1∶200) was used to probe for the VCAM-1 antibody. After three washes in TBS-0.1% BSA, the coverslips were mounted using Vectashield mounting medium containing DAPI nuclear stain. The cells were imaged using an LSM 700 confocal microscope. The image was analyzed for fluorescence signal intensity using MetaMorph software (Molecular Devices, Sunnyvale, CA, USA).

### qRT-PCR

Cells (10^5^ per well) were seeded in a 6-well plate and incubated for 24 hours at 37°C and 5% CO_2_. The cells were then starved in 0.1% serum-EBM-2 for 12 hours followed by incubation for an hour in the presence of different concentrations of compound **2**. The cells were then challenged for 24 hours with 10 ng/ml TNF-α. Following incubation, cells were lysed and RNA was isolated using Trizol reagent (Life Technologies). cDNA was prepared from 80 ng total RNA using random primers and M-MuLV Reverse Transcriptase (New England Biolabs, Ipswich, MA, USA). RT-PCR reactions were set up using the TaqMan Fast Gene Expression Assay Kit according to the manufacturer's instructions. FAM-labeled TaqMan probes for *PTGS2* (Hs00153133_m1), *CCL2* (Hs00234140_m1), *IL8* (Hs00174103_m1), and *TBP* (Hs99999910_m1) genes were used to monitor the expression levels of these genes. The qRT-PCR plate was read in a ViiA™ 7 qPCR system (Life Technologies) and the data were analyzed using the ΔΔC_t_ method. The expression levels of genes were normalized to *TBP* gene expression and calibrated to the DMSO-treated, unstimulated sample.

### Immunoblot

HRECs were seeded at 10^5^ cells/well in a 6-well plate and after 24 hours of incubation at 37°C, cells were serum starved in 0.1% serum-EBM-2 for 8 hours. Cells were then treated with the indicated concentrations of compound **2** for one hour before the addition of 20 ng/ml of TNF-α or 100 ng/ml VEGF. After 20 minutes, cells were lysed in NP-40 Lysis buffer containing 25 mM HEPES pH 7.4, 1% NP-40, 150 mM NaCl, 10% glycerol, 1 mM sodium orthovanadate, 10 mM sodium fluoride, 1 mM sodium pyrophosphate, 1 mM PMSF, 2.5 mg/ml aprotinin, 1 mM pepstatin, and 1 mM leupeptin. Equal amounts of proteins (80 µg), as measured by a Bradford assay, were run on 10% SDS-PAGE, transferred to PVDF membrane, blocked with 5% BSA in TBS-0.05% Tween-20 and immunoblotted with the indicated primary antibodies (1∶1000 in 1% BSA in TBS-0.05% Tween-20) overnight at 4°C. After three washes in TBS-0.05% Tween-20, HRP-conjugated secondary antibodies (1∶5000 in 5% BSA in TBS-0.05% Tween-20) were applied for one hour at room temperature. After three washes, the protein bands were detected and densitized using ECL Prime western blot detection reagent (GE Life Sciences, Piscataway, NJ, USA) and an XRS gel documentation system running Quantity One software (Bio-Rad). Target protein band intensity was normalized to housekeeping gene α-tubulin. For phosphoprotein analysis, normalized signal of each phosphoprotein was expressed relative to the normalized total amount of that protein.

### Statistical Analysis

EdU incorporation, tube formation, apoptosis, immunoblot, and VCAM-1 staining assay data were analyzed by ANOVA with Dunnett's post hoc tests for comparisons between drug treatments and control. All analyses were performed with GraphPad Prism (v. 6.0). A *p*-value<0.05 was considered statistically significant in all tests.

## Results

### Synthesis of SH-11052

To begin to explore synthetic homoisoflavanones as antiangiogenic compounds, we synthesized a regioisomer of compound **1**, the novel homoisoflavanone SH-11052 (**2**; Fig. 1A). Homoisoflavanones consist of a chromanone with a substituted benzyl group at the C3 position in the C ring. Among them, compound **2** is a unique homoisoflavanone comprising dihydroxy at the C5 and C6 positions and a methoxy at the C7 position, respectively, with a 3′-hydroxy-4′-methoxybenzyl group at the C3 position of chromanone. This compound has not previously been reported, although syntheses of similar compounds are known [26]–[30]. The substantial challenge associated with the total synthesis of compound **2** was to uncover three phenolic groups on the C5, C6 (in the A ring) and C3′ (in the B ring) positions. For synthesis of homoisoflavanone **2** and analogs, we envisaged the chromanone ring formation by the treatment of a dihydrochalcone with formaldehyde and a regioselective demethylation among the methoxy groups of the A and B rings.

**Figure 2:**
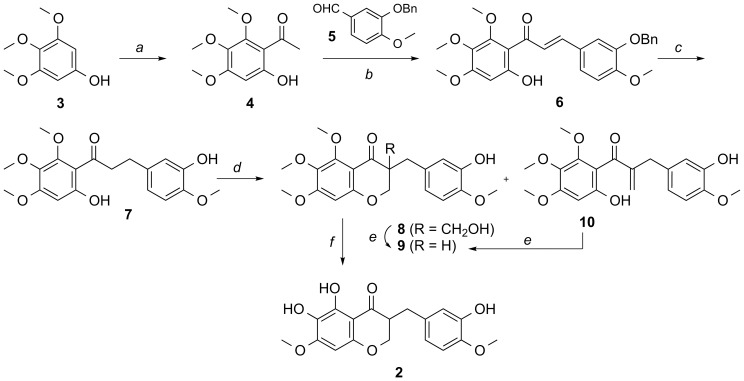
Synthesis of novel homoisoflavanone SH-11052 (2). Reagents and conditions: a) acetic anhydride, BF_3_-OEt_2_; b) **5**, KOH, MeOH, 0°C, 56%; c) Pd/C, HCO_2_Na, HCO_2_H, 60°C, 79%; d) formalin, NaOH, 60°C, 54% for **8**, 10% for **9**, and 15% for **10**; e) K_2_CO_3_, EtOH, 49% from **8**, 72% from **10**; f) TMSI, CHCl_3_, 49%.

As shown in Fig. 2, our approach toward the synthesis of compound **2** commenced with an aldol condensation of the 6′-hydroxy-2′,3′,4′-trimethoxy-acetophenone **4**, which was prepared from 3′,4′,5′-trimethoxyphenol **3** [31] with 3-benzyloxy-4-methoxybenzaldehyde **5** to afford the resulting chalcone **6** in moderate yield. Catalytic hydrogenation of the chalcone **6** under HCO_2_Na and Pd/C afforded the dihydrochalcone **7**. With the dihydrochalcone **7** in hand, the chromanone ring was constructed by hydroxymethylation and cyclization. To this end, the desired chromanone **9** was obtained in good yield by aldol reaction with formaldehyde under basic conditions, and subsequent treatment with K_2_CO_3_ of the concomitant compounds **8** and **10**. Finally, two methyl groups were selectively removed by excess of TMSI (6∼8 equivalents) to give SH-11052 (**2**). Note that our synthetic molecule **2** is a racemate; the configuration at C3 for compound **1** has not been reported. Further experiments are necessary to determine the absolute configuration of the C3 position in compound **1**.

### SH-11052 Inhibits Proliferation of HRECs

**Figure 3:**
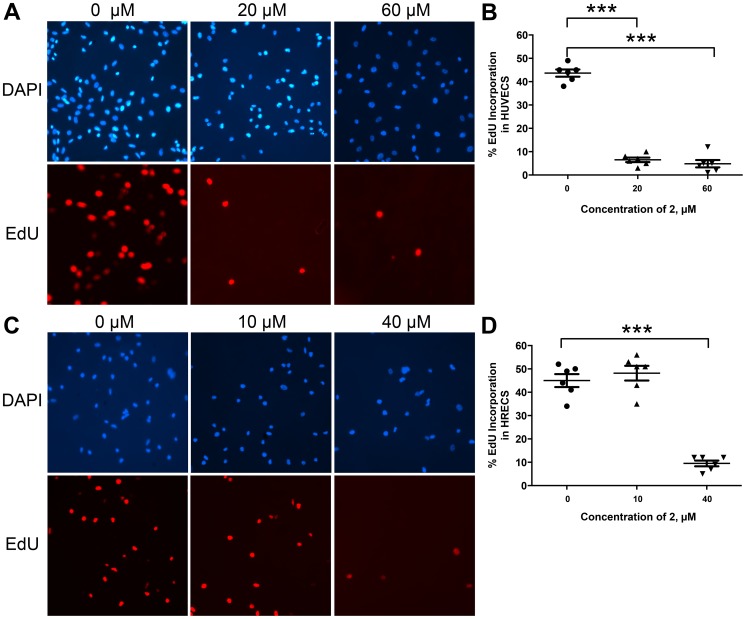
SH-11052 blocks DNA synthesis in endothelial cells. After treatment with the indicated concentrations of SH-11052 (**2**) and an EdU pulse, the HUVECs (A, B) and HRECs (C, D) were stained with DAPI (for nucleus - *blue*) and incorporated EdU (in proliferating cells – *red*) using a Click-iT kit (Life Technologies). The cells were counted from 6 different fields of a coverslip and the percentage of proliferating HUVECs (B) and HRECs (D) was calculated from the ratio of EdU stained cells to DAPI stained cells in each section (dots in the graphs) using ImageJ analysis software. The lines indicate the mean ± SEM and *** indicates *p*<0.0001 (ANOVA with Dunnett's post hoc test). Representative data from three independent experiments.

It has been reported that compound **1** isolated from *C. appendiculata* showed antiproliferative effects with a 50% growth inhibitory (GI_50_) concentration value in the low micromolar range in a HUVEC proliferation assay [21], [23]. In order to test if SH-11052 (**2**) has similar effects, the proliferation of HUVECs induced by complete medium was monitored in the presence of compound **2** in the concentration range of 0.5 nM to 500 µM. As shown in Fig. 1B, compound **2** inhibited HUVEC proliferation in a dose dependent manner with GI_50_ = 18 µM. Once the antiproliferative activity of compound **2** against HUVECs was confirmed, the proliferation of the more disease-relevant HRECs was tested in the presence of compound **2** in the same concentration range. As shown in Fig. 1C, HREC proliferation was significantly inhibited by compound **2** with GI_50_ = 43 µM. In order to confirm the inhibition of cell proliferation, we monitored incorporation of 5-ethynyl-2′-deoxyuridine (EdU) into endothelial cells in the presence of compound **2** (Fig. 3). DNA synthesis in both HRECs and HUVECs was significantly inhibited in a dose dependent manner by this compound.

### SH-11052 Inhibits *In Vitro* Angiogenesis without Inducing Apoptosis

**Figure 4:**
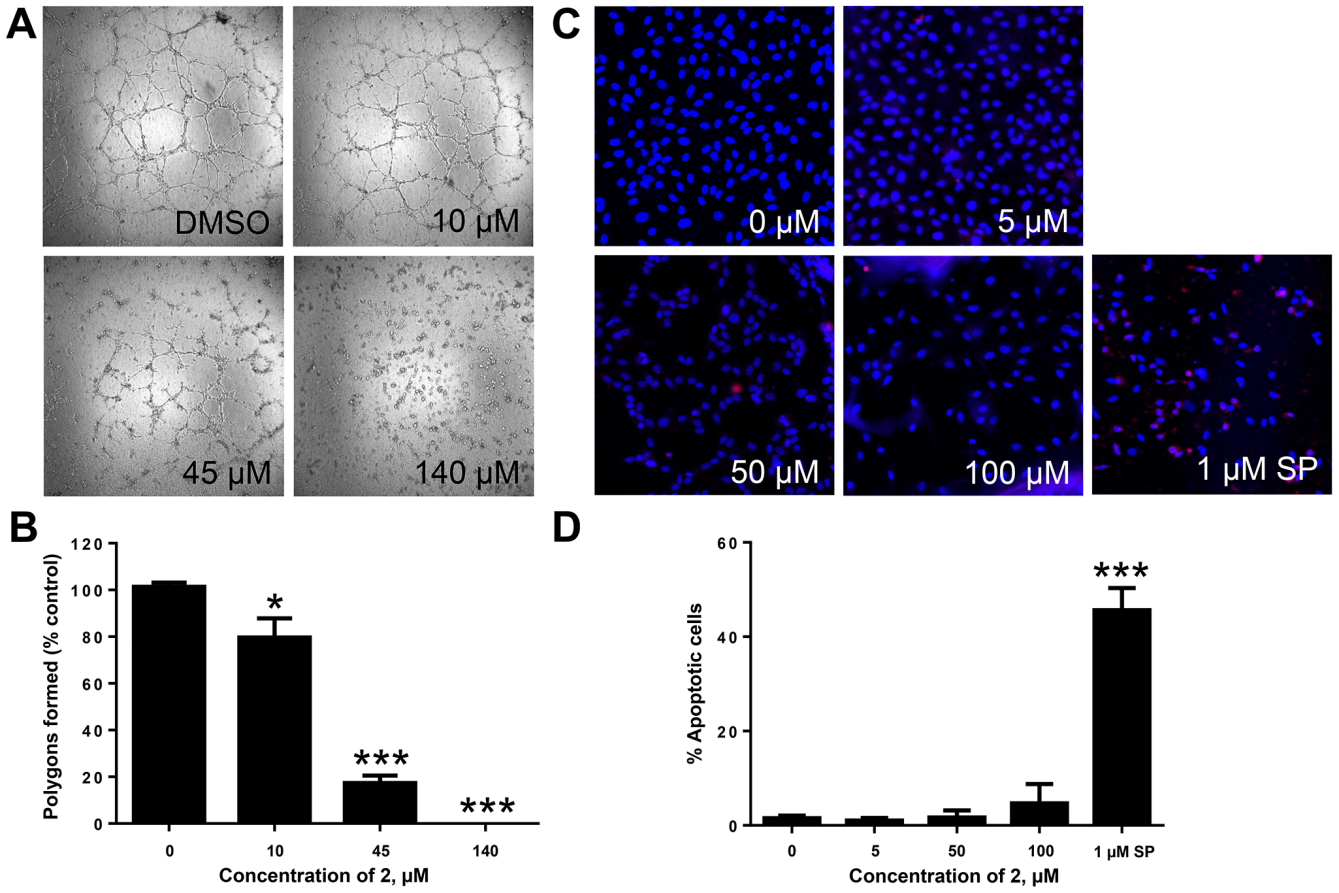
SH-11052 inhibits *in vitro* angiogenesis without causing apoptosis. (A) Tube formation on Matrigel by HRECs in the presence of the indicated concentrations of **2**. (B) Polygons formed (open spaces) were counted. Mean ± SEM of n = 3 wells. *, *p*<0.05; ***, *p*<0.001 compared to DMSO control (ANOVA with Dunnett's post hoc test). (C) HRECs were treated with indicated concentrations of **2** or staurosporine (SP) and stained with DAPI (for nucleus - *blue*) and activated caspase-3 antibody (*red*). (D). Percentage of HRECs undergoing apoptosis was calculated by counting number of caspase (*red*) stained cells compared to total (*blue*) cells using ImageJ software. Mean ± SEM of cells from 3 different sections; representative data from two independent experiments. *** *p*<0.001 compared to DMSO control (ANOVA with Dunnett's post hoc test).

The effect of compound **2** on the angiogenic ability of HRECs was evaluated using an *in vitro* Matrigel tube formation assay. HRECs treated with compound **2** showed a significant reduction in their tube formation ability as compared to DMSO treated samples (Fig. 4A). In the presence of compound **2** at the GI_50_ value, there was a significant reduction in tube formation and the network of tubes was disrupted (polygon spaces in Fig. 4A) and at 120 µM the tube formation ability was completely abolished (Fig. 4B). However, even at 100 µM, compound **2** caused negligible apoptosis of HRECs as determined by cleaved caspase-3 staining (Fig. 4C and 4D).

### SH-11052 Inhibits the TNF-α Mediated NF-κB Pathway

**Figure 5:**
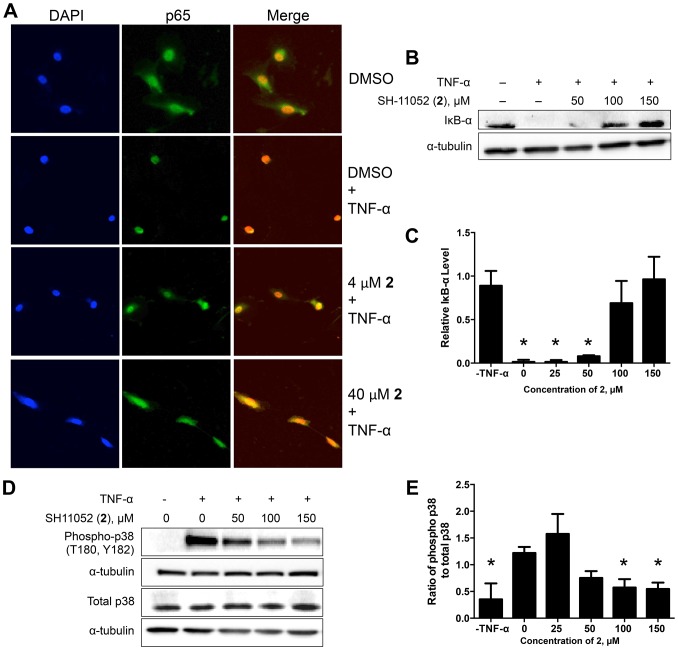
SH-11052 inhibits TNF-α mediated NF-κB signaling. (A) After treating HRECs with the indicated concentrations of **2**, p65 (*green*) was detected by immunofluorescence and nuclei (*blue*) stained with DAPI. Representative data from three independent experiments. (B) The protein levels of IκB-α were measured after TNF-α treatment in the presence of the indicated concentrations of compound **2** by immunoblot. (D) The phosphorylation level of p38 MAPK in HRECs stimulated with TNF-α was monitored in the presence of the indicated concentrations SH-11052 by immunoblot. (C & E) Densitometry was performed using Quantity One software and analyzed using GraphPad Prism. The lines indicate the mean ± SEM of three biological replicates and * indicates *p*<0.05 compared to TNF-α treatment (ANOVA with Dunnett's post hoc test.

After establishing the anti-angiogenic activity of compound **2**, we examined the mechanistic details of its activity in HRECs. As inflammation plays a crucial role in pathological angiogenesis [32]–[35], we examined the effect of compound **2** on inflammatory signaling in endothelial cells. HRECs were treated with different concentrations of compound **2** and then induced with TNF-α, a known pro-inflammatory cytokine and inducer of NF-κB. Since NF-κB exerts its transcriptional activity in the nucleus, blockade of stimulus-induced nuclear translocation of NF-κB is an indication of NF-κB pathway inhibition [36]. The nuclear translocation of NF-κB upon TNF-α stimulation was inhibited by compound **2** in a dose dependent manner as monitored by immunofluorescence (Fig. 5A). IκB-α is an inhibitory protein that binds to NF-κB and prevents its nuclear translocation. Upon TNF-α stimulation, IκB-α is phosphorylated and degraded, freeing NF-κB for nuclear translocation [37]. In the presence of compound **2**, the TNF-α-mediated degradation of IκB-α was significantly decreased in a dose dependent manner, further indicating that compound **2** is inhibiting NF-κB signaling (Fig. 5B–C). In order to confirm inhibition of the TNF-α pathway, we monitored the activating phosphorylation of p38 mitogen activated protein kinase (MAPK), an important downstream target of the TNF-α pathway involved in cytokine induced cell proliferation [38]. Compound **2** inhibited phosphorylation of p38 MAPK in a dose dependent manner (Fig. 5D–E).

To confirm that NF-κB pathway inhibition can lead to decreased proliferation of HRECs, we treated these cells with NF-κB pathway specific inhibitors BAY 11-7082, which inhibits TNF-α induced phosphorylation of IκB-α [39], and caffeic acid phenethylester (CAPE), which can block NF-κB-mediated transcription [40]. Both compounds inhibited proliferation of HRECs in a dose dependent manner with GI_50_ values of 4 µM for BAY 11-7082 and 36 µM for CAPE.

### SH-11052 Decreases Levels of NF-κB Targets

**Figure 6:**
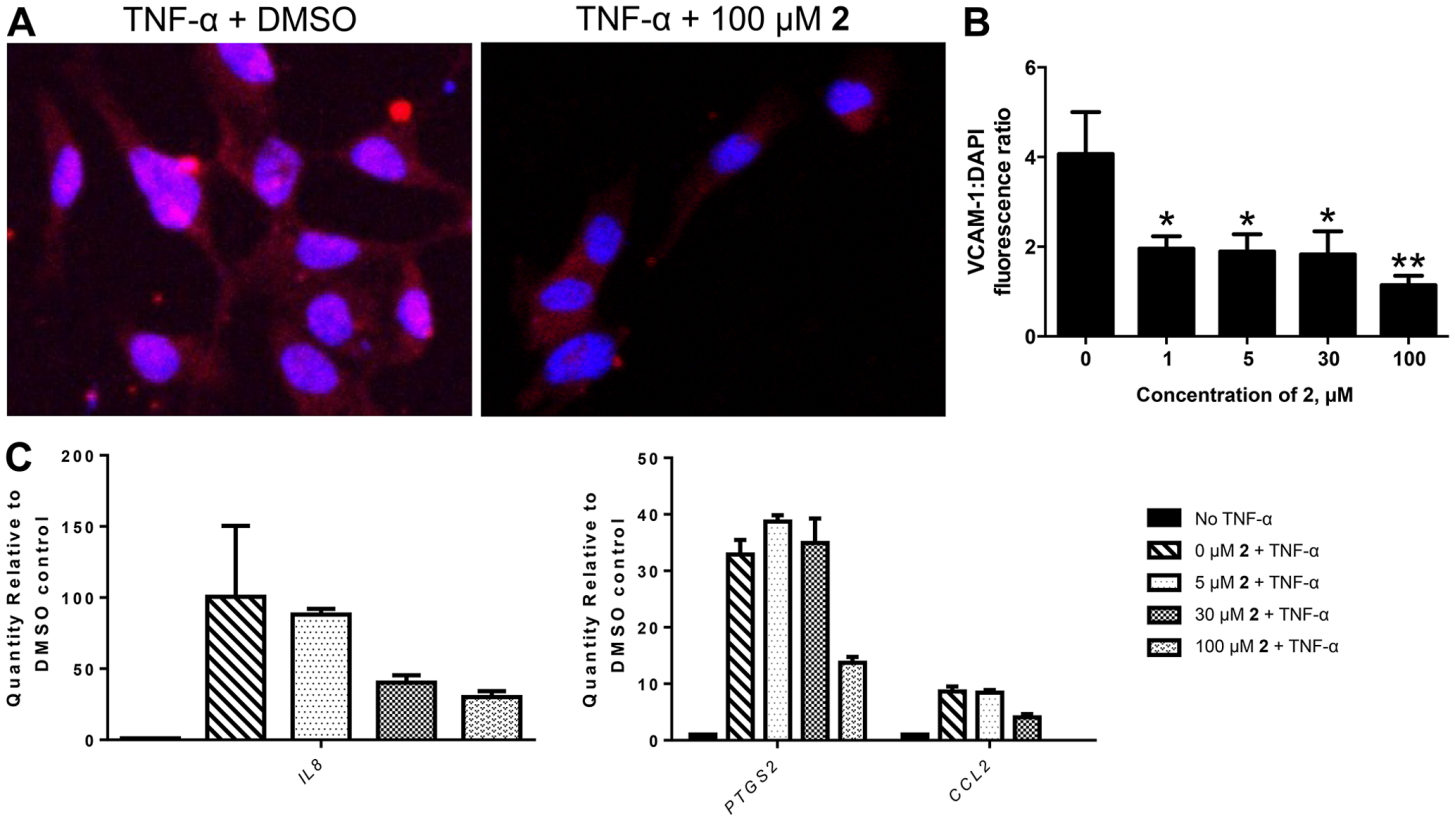
SH-11052 decreases the expression of NF-κB target genes. (A) Endothelial activation marker VCAM-1 (*red*) was detected by immunofluorescence in HRECs exposed to TNF-α ± **2**. (B) MetaMorph fluorescence intensity analysis of VCAM-1 staining in the presence of TNF-α and the indicated concentrations of **2**, mean±SEM of n = 5 fields; *, *p*<0.05 **, *p*<0.01 compared to DMSO control (ANOVA with Dunnett's post hoc test); representative data from two independent experiments. (C) qRT-PCR using TaqMan probes showed that mRNA levels of NF-κB target genes *IL8* (interleukin-8) (*left panel*), *CCL2* (MCP-1) and *PTGS2* (COX2) (*right panel*), all induced by TNF-α, were decreased in the presence of **2** in a dose dependent manner. Note different y-axis scales. Mean ± SEM of n = 3 replicates shown; representative data from two independent experiments.

We confirmed NF-κB pathway inhibition by monitoring the expression of NF-κB induced genes in the presence of compound **2** (Fig. 6). VCAM-1 is a cell adhesion molecule specifically expressed on endothelial cells and its expression is induced by NF-κB upon TNF-α signaling [41], [42]. We monitored the expression of VCAM-1 in HRECs using immunofluorescence with increasing concentrations of compound **2**. There was a significant dose-dependent decrease in VCAM-1 protein expression in the presence of compound **2** (Fig. 6A & 6B). Similarly the mRNA expression of the pro-inflammatory molecules *IL8*, *PTGS2* (COX2) and *CCL2* (MCP-1), inducible by NF-κB, were decreased in the presence of compound **2**, as monitored by qRT-PCR (Fig. 6C).

### SH-11052 Inhibits VEGF-mediated Activation of PI3K/Akt Signaling:

**Figure 7:**
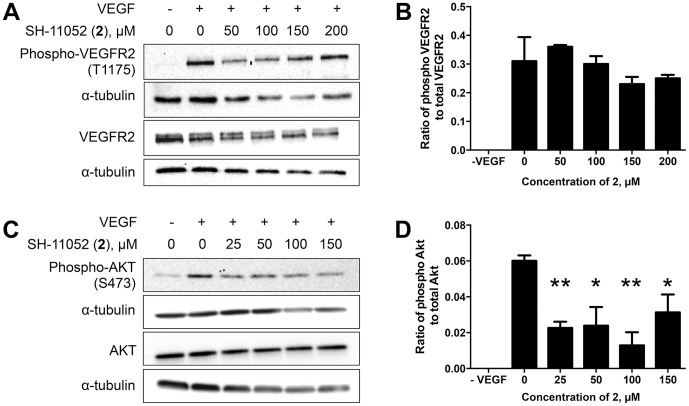
SH-11052 inhibits VEGF mediated Akt signaling. Phosphorylation of VEGFR2 (A) and Akt (C) was monitored in HRECs upon VEGF stimulation in the presence of varying concentrations of **2**. (B & D) Densitometry was performed using Quantity One software and analyzed using GraphPad Prism. The lines indicate the mean ± SEM of three biological replicates, * indicates *p*<0.05 and ** indicates *p*<0.01 compared to VEGF treatment alone (ANOVA with Dunnett's post hoc test).

As VEGF signaling is a major contributor to angiogenesis, we tested if compound **2** can inhibit VEGF signaling along with inflammation induced TNF-α signaling. Upon VEGF stimulation, VEGF receptor 2 (VEGFR2) autophosphorylates, leading to activation of the PI3K/Akt pathway [43]. Compound **2** did not inhibit phosphorylation of VEGFR2 but inhibited activation of the downstream Akt in HRECs (Fig. 7). Since TNF-α signaling also feeds through Akt to IKKα [44], these results suggest that compound **2** might act at the level of PI3K or Akt to block both VEGF and TNF-α signaling.

## Discussion

In pathological ocular neovascularization such as that observed in ROP, DR and AMD, there is an increase in the levels of VEGF [45], [46]. Along with VEGF, inflammation plays a crucial role in pathological angiogenesis, as we and others have shown [11], [35], [45], [47]. This suggests that pathologic vessel growth as observed in ocular diseases is not only under control of VEGF [48], but also intimately linked with inflammation leading to endothelial activation [34], [42]. Of the several cell signal transduction pathways studied, NF-κB pathways play an important role in inducing the expression of pro-angiogenic genes such as *VEGF*, *VEGFR*, *IL8*, *VCAM1*, *CCL2*, and *PTGS2* after activation through TNF-α signaling [33], [45], [49]. Based on the clinical evidence that anti-VEGF antibody therapy led to arterial thromboembolic complications and suggested a role for endothelial damage and inflammation [50], a combination of anti-VEGF and anti-inflammatory therapy might be more beneficial in treating neovascular eye disease than monotherapy. In favor of this hypothesis, in ROP experimental models, a combination of inhibitors of different pathways was found to be highly significant in blocking new vessel formation [51].

At present, the current antiangiogenic biologics only target VEGF signaling at the level of the receptor. Therefore, the development of drugs targeting inflammatory signals provides an opportunity for new, combination anti-angiogenic therapies. Developing small molecules to include in such cocktails is thus of significant interest and development of drugs which can inhibit multiple angiogenic pathways would greatly increase the efficacy of therapies. Towards this goal we have pursued SH-11052 as a small molecule inhibitor of retinal neovascularization, building on previous reports of the efficacy of a related homoisoflavanone (**1**) in animal models of ROP and choroidal neovascularization [23], [24].

In the present study we have used a novel method based on a chalcone intermediate and a regioselective demethylation (Fig. 2) to synthesize the novel homoisoflavanone **2**. This methodology adds to the diversity of homoisoflavanones that are synthetically tractable and will readily allow synthesis of novel analogs in future. We have confirmed that our synthetic SH-11052 has similar effects on HUVECs to the natural-source compound **1** (Fig. 1B). GI_50_ of the natural product **1** was reported as 0.5 µg/mL, or approximately 1.5 µM [21].

Endothelial cells from different tissues have different gene expression patterns suggesting different physiological effects [52]. HUVECs are macrovascular endothelial cells which do not have specific relevance to the microvascular endothelial cells that are present in retinal capillaries of the eye. Therefore, we tested SH-11052 in HRECs, where it proved similarly potent as an anti-proliferative molecule (Fig. 1C), albeit at higher GI_50_, consistent with the hypothesis that microvascular endothelial cells differ from macrovascular endothelial cells. SH-11052 blocks proliferative progression through DNA synthesis in both HUVECs and HRECs (Fig. 3). This is consistent with the documented G_2_/M phase cell cycle arrest induced by **1** in HUVECs [24]. We have also demonstrated the *in vitro* anti-angiogenic activity of **2** in a Matrigel HREC tube formation assay, similar to the effects of **1** in HUVECs [24].

The novel anti-angiogenic mechanism of homoisoflavanones remains largely unexplored. In HUVECs, compound **1** induced expression of p21^WAF1^ (*CDKN1A*), an inhibitor of the cyclin-dependent kinase Cdc2 (*CDK1*), which in turn is downregulated by compound **1** [24]. Homoisoflavanone **1** also blocked prostaglandin synthesis from arachidonic acid in a microsome assay, without marked effects on function of cyclooxygenases 1 and 2 as purified enzymes [53]. In keratinocytes, compound **1** inhibited the nuclear translocation of NF-κB under ultraviolet light-induced inflammatory conditions, suggesting a role of the compound in modulating inflammatory signals in these cells [54]. In this context, compound **1** also decreased phosphorylation of the MAPKs Jun N-terminal kinase (JNK), p38 MAPK, and ERK.

We examined if the activity of SH-11052 in HRECs may likewise be mediated through modulation of inflammatory signals. As NF-κB is the principal mediator of inflammation induced signals [55], we monitored NF-κB activation upon TNF-α stimulation in the absence or presence of compound **2** in HRECs. NF-κB is a transcription factor sequestered in the cytoplasm by association with IκB-α protein. Upon TNF-α stimulation, IκB-α is phosphorylated and degraded by the proteasome, releasing free NF-κB. The free NF-κB is then translocated into the nucleus and aids in the transcription of its target genes. Hence monitoring the protein levels of IκB-α and nuclear translocation of NF-κB upon TNF-α treatment are measures of the activation of the NF-κB pathway by inflammatory signals [55]. Indeed, we show that IκB-α degradation and nuclear translocation of NF-κB in HRECs are inhibited by compound 2 (Fig. 5). Furthermore, compound **2** also inhibited the expression of NF-κB inducible pro-angiogenic and pro-inflammatory genes (Fig. 6), suggesting a role for compound **2** in the inhibition of inflammation-induced pro-angiogenic signaling in HRECs.

SH-11052's suppressive effects on expression of *IL8* and *PTGS2* in HRECs are consistent with the observed effects of the natural product **1** in keratinocytes. To our knowledge, we show for the first time an effect of a homoisoflavanone on the endothelial activation marker and NF-κB target, VCAM-1, and on the inflammatory marker *CCL2*. Thus, the data presented here are consistent with a function for compound **2** as an inhibitor of NF-κB signaling in HRECs. NF-κB has previously been implicated in pathological ocular angiogenesis [56], [57] and we further confirmed that two known NF-κB pathway inhibitors, BAY 11-7082 [39] and CAPE [40], had antiproliferative effects on HRECs.

This assertion of an NF-κB-dependent role for compound **2** can integrate others' findings regarding the activities of the related compound **1** in other cell types as well. bFGF can act by signaling through phospholipase Cγ1 [58], [59], which activates protein kinase C (PKC) α via diacylglycerol. In turn, PKCα binds and activates IKKα, which phosphorylates and inactivates I-κB [60]. Thus, blockade of this pathway would inhibit cellular responses to bFGF, as seen with compound **1** in HUVECs [21]. NF-κB is a major transcription factor for inflammatory cytokines, and also for transcription of *PTGS2* [61], consistent with the negative transcriptional effects of compound **1** on these genes and consequent inhibition of prostaglandin production in keratinocytes. Mitochondrial superoxide dismutase (*SOD2*) transcription is also activated by NF-κB [62], potentially explaining increased reactive oxygen species generation after NF-κB inhibition mediated by compound **1** [54]. Moreover, blockade of the NF-κB pathway can deactivate MAPKs, as seen with compound **1** [54] in keratinocytes and compound **2** in HRECs (Fig. 5). This may occur via sequestration of the MAPK kinase kinase TPL2 (tumor progression locus 2) by (inactive) p105^NFKB1^ [63]. Finally, NF-κB inhibition can lead to upregulated p53-mediated transcription [64], which could increase levels of p21^WAF1^ and decrease Cdc2 expression in response to treatment with compound **1** as previously seen in HUVECs [24]. A role for both compounds **1** and **2** in the NF-κB pathway is also consistent with possible mechanisms for related isoflavones [65], and NF-κB pathway inhibition has previously been shown to block pathogenic ocular neovascularization [56].

Compound **2** may be acting at the level of PI3K or Akt, since compound **2** can inhibit VEGF-induced Akt phosphorylation, but not VEGFR2 phosphorylation (Fig. 7), and since PI3K/Akt serves as a point of integration of both VEGF and TNF signaling [44]. As a post-receptor VEGFR signaling inhibitor and TNF-α pathway inhibitor, compound **2** may serve as a lead molecule for targeting multiple proangiogenic pathways to improve the efficacy of treatment. Development of such molecules which target both VEGF and other inflammation induced proangiogenic signaling pathways might help in replacing or reducing the dose of anti-VEGF biologics. Future studies identifying the direct molecular interacting partners of homoisoflavanones in HRECs will be crucial for elucidating their precise anti-angiogenic mechanism, as well as optimizing the structures of these molecules prior to future therapeutic use, via a target-based drug discovery approach.

In summary, we have synthesized a novel homoisoflavanone **2** and demonstrated its anti-angiogenic activity in retinal endothelial cells, and provided partial characterization of its molecular mechanism. Given SH-11052's anti-angiogenic properties, it will be important to fully elucidate the molecular mechanism of action of this compound. Our synthetic method also enables structure-activity profiling to obtain more potent anti-angiogenic molecules that can be tested in animal models of ocular neovascularization, both alone and in combination therapies.

## Supporting Information

File S1 Contains **Methods S1**, Complete synthetic methodology and chemical characterization for SH-11052 and intermediates (PDF); and **Figure S1**, Structural assignment of SH-11052 (**2**) by NOESY and HMBC.
(PDF)
