# Supplementary material for: Synthesis and Mechanistic Studies of a Novel Homoisoflavanone Inhibitor of Endothelial Cell Growth
Source: PLoS One. 2014 Apr 21;9(4):e95694. doi: 10.1371/journal.pone.0095694 (PMC3994091; doi:10.1371/journal.pone.0095694)
Supplement: File S1 — Contains Methods S1, Complete synthetic methodology and chemical characterization for SH-11052 and intermediates (PDF); and Figure S1, Structural assignment of SH-11052 (2) by NOESY and HMBC. (PDF) [file pone.0095694.s001.pdf]

Basavarajappa HD, Lee B, Fei X, Lim D, Callaghan B, Mund JA, Case J, Rajashekhar G, Seo S-Y, Corson TW. Synthesis and mechanistic studies of a novel homoisoflavanone inhibitor of endothelial cell growth.

SUPPLEMENTAL METHODS S1

Experimental Section

General

All starting materials and reagents were obtained commercially and were used without further purification. Tetrahydrofuran was distilled from sodium benzophenone ketyl. Dichloromethane and acetonitrile were freshly distilled from calcium hydride. All solvents used for routine product isolation and chromatography were of reagent grade and glass distilled. Reaction flasks were dried at 100°C before use, and air- and moisture-sensitive reactions were performed under argon. Flash column chromatography was performed using silica gel 60 (230−400 mesh, Merck) with the indicated solvents. Thin-layer chromatography was performed using 0.25 mm silica gel plates (Merck). Mass spectra were obtained using a Waters Auto Purification instrument, and high resolution mass spectra were obtained using a JEOL JMS-AX 505WA unit. ^{1}H and ^{13}C spectra were recorded on either a Bruker AVANCE III 400MHz, or a Bruker AVANCE III 600MHz spectrometer as solutions in deuteriochloroform ($CDCl_3$) and methanol-d4. ^{1}H NMR data were reported in the order of chemical shift, multiplicity (s, singlet; d, doublet; t, triplet; m, multiplet and/or multiple resonances), number of protons, and coupling constant (J) in hertz (Hz).

*1-(6-hydroxy-2,3,4-trimethoxyphenyl)ethanone (**4**)*

To an acetic anhydride (2 mL) solution of 3,4,5-trimethoxyphenol (**3**) (1.2 g, 6.6 mmol), BF_3-Et_2O (0.07 mL) was added at 0°C. After stirring at 60°C for 3 h, the reaction mixture was diluted with ethyl acetate and the reaction mixture was cooled to ca. 0°C for 2 h and the crystallized cake filtered with ethyl acetate. H_2O (10 mL) and Et_3N (1 mL) were added. After stirring for 1 h at room temperature, the reaction mixture was diluted with ethyl acetate and the organic phase was washed with water and brine, dried over $MgSO_4$ and concentrated under reduced pressure. The residue was purified by flash column chromatography (Ethyl acetate / *n*-hexanes = 1 : 1) to afford the methyl ketone (**4**) (1.4 g, 95%). The compound **4** was reported. See Supplemental Ref [1].

*(E)-3-(3'-benzyloxy-4'-methoxyphenyl)-1-(6-hydroxy-2,3,4-trimethoxyphenyl)prop-2-en-1-one (**6**)*

To a solution of methyl ketone (**4**) (1.5 g, 6.5 mmol) in MeOH (10 mL) was added 3-benzyloxy-4-methoxybenzaldehyde (**5**) (2.0 g, 8.0 mmol) and KOH (1.5 g, 25 mmol) at 0°C, then warmed to rt. To the reaction mixture was stirred at 35°C for 72 h followed by the addition of water and dilution with CH_2Cl_2. The organic layer was washed with water and brine, dried over $MgSO_4$, and concentrated in vacuo. The residue was purified by silica gel column chromatography (Ethyl acetate / *n*-hexane = 1 : 3) to afford 3'-benzyloxy-4'-methoxychalcone (**6**) (1.6 g, 56%). ^{1}H-NMR (600 MHz, $CDCl_3$) δ 13.7 (s, 1H), 7.74 (s, 2H), 7.47 (d, 2H, J = 7.2 Hz); 7.40 (t, 2H, J = 7.2 Hz); 7.33 (d, 1H, J = 7.2 Hz); 7.24 (dd, 1H, J = 8.4 and 2.4 Hz); 7.17 (d, 1H, J = 1.2 Hz); 6.92 (d, 1H, J = 8.4 Hz); 6.28 (s, 1H), 5.22 (s, 2H), 3.94 (s, 3H), 3.89 (s, 3H), 3.84 (s, 3H), 3.83 (s, 3H); ^{13}C-NMR

(150 MHz, $CDCl_3$) δ 192.7, 162.6, 159.9, 154.9, 151.9, 148.3, 143.5, 136.7, 135.2, 128.7, 128.2, 128.0, 127.2, 124.2, 123.5, 113.0, 111.5, 108.7, 96.6, 71.12, 61.9, 61.32, 56.12, 29.7; LRMS (ESI) *m/z* 315 (M+H).

*1-(6-hydroxy-2,3,4-trimethoxyphenyl)-3-(3'-hydroxy-4'-methoxyphenyl)propan-1-one (**7**)*

3'-(benzyloxy)-4'-methoxychalcone (**6**) (850 mg, 1.9 mmol) in isopropanol (10 mL) was added HCO_2Na (513 mg, 7.5 mmol), Pd/C (195 mg, 1.8 mmol) and HCO_2H (1 mL) at 0°C. The reaction mixture was stirred at 60°C for 6 h. The mixture was filtered through a short pad of silica gel. After the filtrate was concentrated in vacuo, purification of the residue via flash column chromatography on silica gel (Ethyl acetate / *n*-hexane = 1 : 3) afforded dihydrochalcone (**7**) (517 mg, 79%). ^{1}H-NMR (400 MHz, $CDCl_3$) δ 13.38 (s, 1H), 6.82 (d, 1H, *J* = 8.28 Hz); 6.73 (s, 2H), 6.21 (s, 1H), 5.53 (s, 1H), 3.93 (s, 3H), 3.85 (d, 6H, *J* = 1.96 Hz); 3.74 (s, 3H), 3.31 (m, 2H), 2.94 (d, 2H, *J* = 7.8 Hz); ^{13}C-NMR (100 MHz, $CDCl_3$) δ 204.8, 161.7, 159.8, 155.0, 146.3, 143.7, 134.6, 133.3, 120.7, 114.2, 111.1, 108.1, 96.1, 61.0, 60.9, 55.9, 55.7, 45.1, 30.2; LRMS (ESI) *m/z* 317 (M+H).

*3-(3'-hydroxy-4'-methoxybenzyl)-3-(hydroxymethyl)-5,6,7-trimethoxychroman-4-one (**8**) and 1-(6-hydroxy-2,3,4-trimethoxyphenyl)-2-(3'-hydroxy-4'-methoxybenzyl)prop-2-en-1-one (**10**)*

The dihydrochalcone (**7**) (700 mg, 1.9 mmol) was dissolved in 50% aqueous NaOH (0.96 mL), H_2O (3.8 mL) and stirred with formalin (0.16 mL, 5.8 mmol) at 60°C for 3h. After stirring for 3 h, the reaction mixture was diluted with ethyl acetate and washed with NH_4Cl and brine, dried over $MgSO_4$ and concentrated under reduced pressure. The residue was purified by flash column chromatography on silica gel (Ethyl acetate / *n*-hexane = 1 : 2) to afford a mixture of compound **8** (383 mg, 54%), **9** (71 mg, 10%) and **10** (106 mg, 15%), respectively. For compound **8**, ^{1}H-NMR (400 MHz, $CDCl_3$) δ 6.83-6.80 (m, 2H), 6.75-6.73 (m, 1H), 6.28 (s, 1H), 5.78 (bs, 1H), 4.04-4.03 (m, 2H), 3.91 (s, 3H), 3.88 (s, 3H), 3.84 (s, 3H), 3.79 (s, 2H), 3.58-3.50 (m, 2H), 3.21 (bs, 1H), 2.98 (d, 1H, *J* = 13 Hz); 2.85 (d, 1H, *J* = 14 Hz); ^{13}C-NMR (100 MHz, $CDCl_3$) δ 196.1, 159.7, 159.4, 154.5, 146.3, 144.5, 137.5, 126.7, 123.3, 114.1, 113.0, 107.8, 95.9, 69.6, 62.2, 61.4, 61.2, 56.0, 55.8, 49.9, 34.8. LRMS (ESI) *m/z* 405 (M+H); For compound **10**, ^{1}H-NMR (400 MHz, $CDCl_3$) δ 11.7 (s, 1H), 7.29 (s, 1H), 7.18 (s, 1H), 6.79 (d, 1H, *J* = 7.8 Hz); 6.67-6.65 (m, 2H), 6.19 (s, 1H), 5.44 (s, 1H), 5.10 (s, 1H), 4.96 (s, 1H), 3.82 (s, 3H), 3.79 (s, 3H), 3.71 (s, 3H), 3.66 (s, 3H), 3.56 (s, 2H); ^{13}C-NMR (100 MHz, $CDCl_3$) δ 201.7, 160.6, 160.2, 151.8, 146.4, 144.1, 134.9, 129.7, 128.3, 122.2, 115.3, 114.2, 112.0, 108.3, 95.9, 61.4, 61.0, 56.1, 55.8, 38.6; LRMS (ESI) *m/z* 375 (M+H).

*3-(3'-hydroxy-4'-methoxybenzyl)-5,6,7-trimethoxychroman-4-one (**9**)*

The compound **8** (100 mg, 0.25 mmol) was dissolved in ethanol (2 mL), and stirred with K_2CO_3 (54 mg, 0.49 mmol) at 90°C for 3 h. After stirring for 3 h, the reaction mixture was diluted with ethyl acetate and washed with 1 N HCl and brine, dried over $MgSO_4$ and concentrated under reduced pressure. The residue was purified by flash column chromatography on silica gel (Ethyl acetate / *n*-hexane = 1 : 2) affording 5,6,7-trimethoxychromanone (**9**) (45 mg, 49%). ^{1}H-NMR (400 MHz, $CDCl_3$) δ 7.24 (s, 1H), 6.83 (d, 1H, *J* = 7.8 Hz); 6.71 (d, 2H, *J* = 1.9 Hz); 6.23 (s, 1H), 5.53 (s, 1H), 4.23 (m, 1H), 4.10 (m, 1H), 3.91 (s, 3H), 3.85 (d, 6H, *J* = 1.9 Hz); 3.79 (s, 3H), 3.16 (m, 1H), 2.70 (m, 1H), 2.63 (m, 1H); ^{13}C-NMR (100 MHz, $CDCl_3$) δ 191.3, 159.6, 159.2, 154.4, 146.5,

144.2, 137.4, 130.2, 121.8, 114.3, 111.4, 108.6, 95.9, 69.0, 61.5, 61.2, 56.0, 55.9, 48.5, 32.5; LRMS (ESI) *m/z* 375 (M+H). From the compound **10** (100 mg, 0.27 mmol), the same reaction condition afforded 5,6,7-trimethoxychromanone (**9**) (72 mg, 72%).

*SH-11052 (**2**)*

To a solution of 5,6,7-trimethoxychromanone (37 mg, 0.10 mmol) in $CHCl_3$ (1 mL) was added TMSI (113 μL, 0.80 mmol) at 0°C and the reaction mixture was heated at 60°C for 4 h. The mixture was concentrated in vacuo. The residue was purified by flash column chromatography on silica gel (Ethyl acetate / *n*-hexane = 1 : 1) to afford SH-11052 (**2**) (17 mg, 49%). ^{1}H-NMR (600 MHz, CD_3OD) δ 6.85 (d, 1H, J = 8.4 Hz), 6.70 (d, 1H, J = 2.4 Hz), 6.67 (dd, 1H, J = 2.4 and 8.4 Hz), 6.13 (s, 1H), 4.25 (dd, 1H, J = 4.2 and 11.4 Hz), 4.09 (dd, 1H, J = 7.8 and 11.4 Hz), 3.87 (s, 3H), 3.82 (s, 3H), 3.08 (dd, 1H, J = 4.8 and 13.8 Hz), 2.84-2.79 (m, 1H), 2.64 (dd, 1H, J = 10.2 and 13.8 Hz); ^{1}H-NMR (600 MHz, $CDCl_3$) δ 11.7 (s, 1H), 6.78 (d, 1H, J = 2.4 Hz), 6.78 (d, 1H, J = 9.6 Hz), 6.67 (dd, 1H, J = 2.4 and 8.4 Hz), 6.02 (s, 1H), 5.60 (s, 1H), 5.02 (s, 1H), 4.25 (dd, 1H, J = 4.2 and 11.4 Hz), 4.10 (dd, 1H, J = 7.8 and 11.4 Hz), 3.88 (s, 3H), 3.85 (s, 3H), 3.15 (dd, 1H, J = 4.8 and 13.8 Hz), 2.82-2.78 (m, 1H), 2.64 (dd, 1H, J = 10.8 and 14.4 Hz); ^{13}C-NMR (150 MHz, CD_3OD) δ 200.7, 157.6, 157.6, 150.4, 148.0, 147.8, 132.4, 128.7, 121.4, 117.1, 113.0, 103.5, 92.25, 70.63, 56.82, 56.58, 33.26; ^{13}C-NMR (150 MHz, $CDCl_3$) δ 200.7, 158.0, 156.6, 150.1, 147.7, 147.4, 133.0, 129.2, 122.6, 117.1, 112.8, 104.4, 93.0, 71.4, 58.3, 58.0, 48.8, 34.1; LRMS (EI) *m/z* 346 (M^+); HRMS (EI) *m/z* 346.1057 (M^+) [calc. $C_{18}H_{18}O_7$ 346.1053].

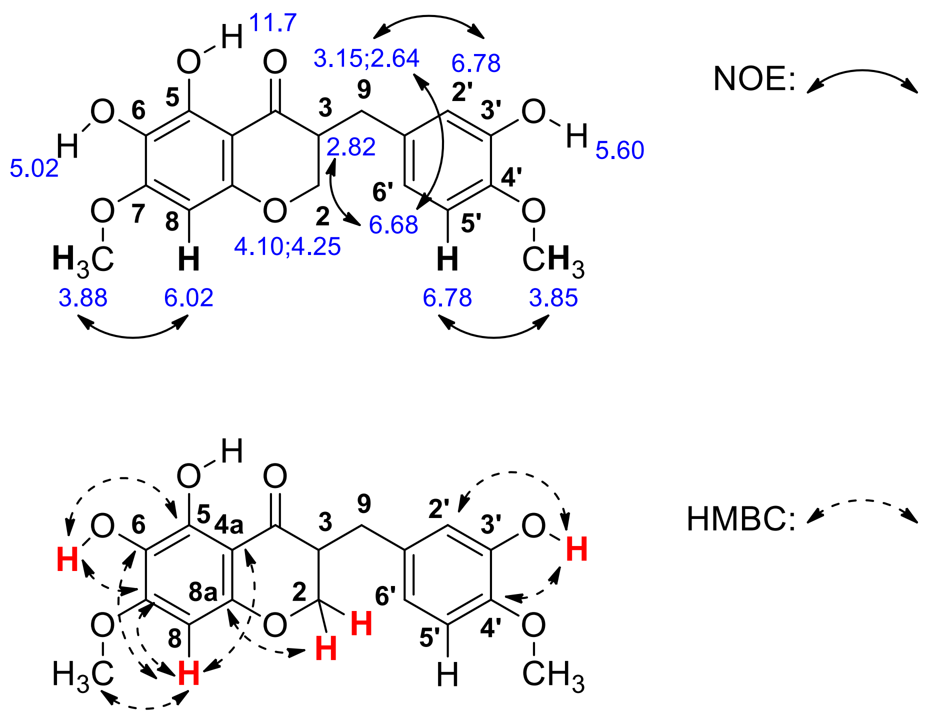

Figure S1. Structural assignment of SH-11052 (**2**) by NOESY and HMBC. (Blue color: chemical shift of ^{1}H-NMR)

Supplemental Reference

1. Chen DZ, Yang J, Yang B, Wu YS, Wu T (2010) Total synthesis of baicalein. J Asian Nat Prod Res 12: 124-128.

#6

1H 1D

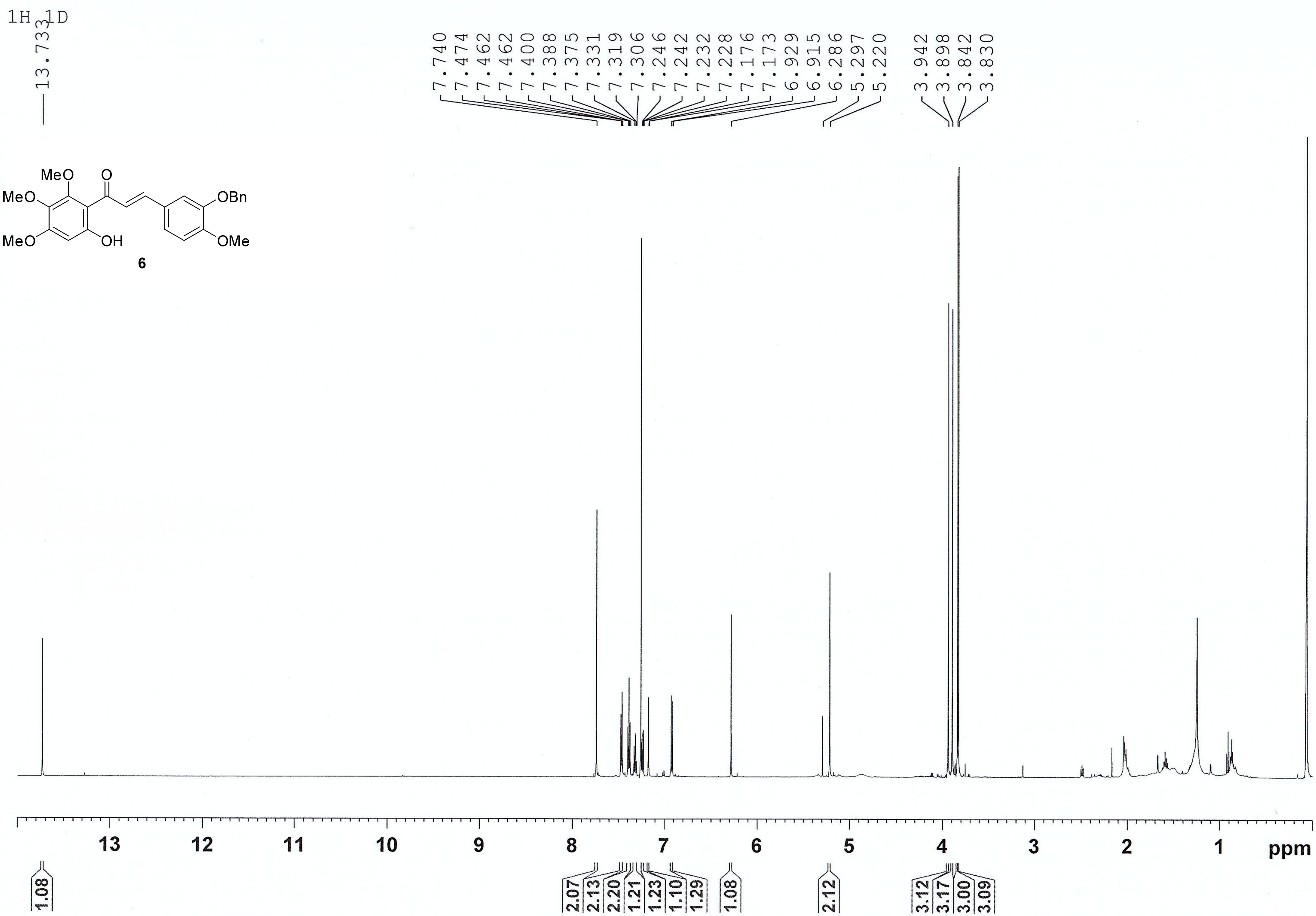

MeO
O
MeO
OBn
MeO
OH
OMe
6
13.733
7.740
7.474
7.462
7.462
7.400
7.388
7.375
7.331
7.319
7.306
7.246
7.242
7.232
7.228
7.176
7.173
6.929
6.915
6.286
5.297
5.220
3.942
3.898
3.842
3.830
13
12
11
10
9
8
7
6
5
4
3
2
1
ppm
1.08
2.07
2.13
2.20
1.21
1.23
1.10
1.29
1.08
2.12
3.12
3.17
3.00
3.09

#6

Carbon 1D

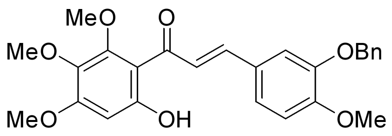

6

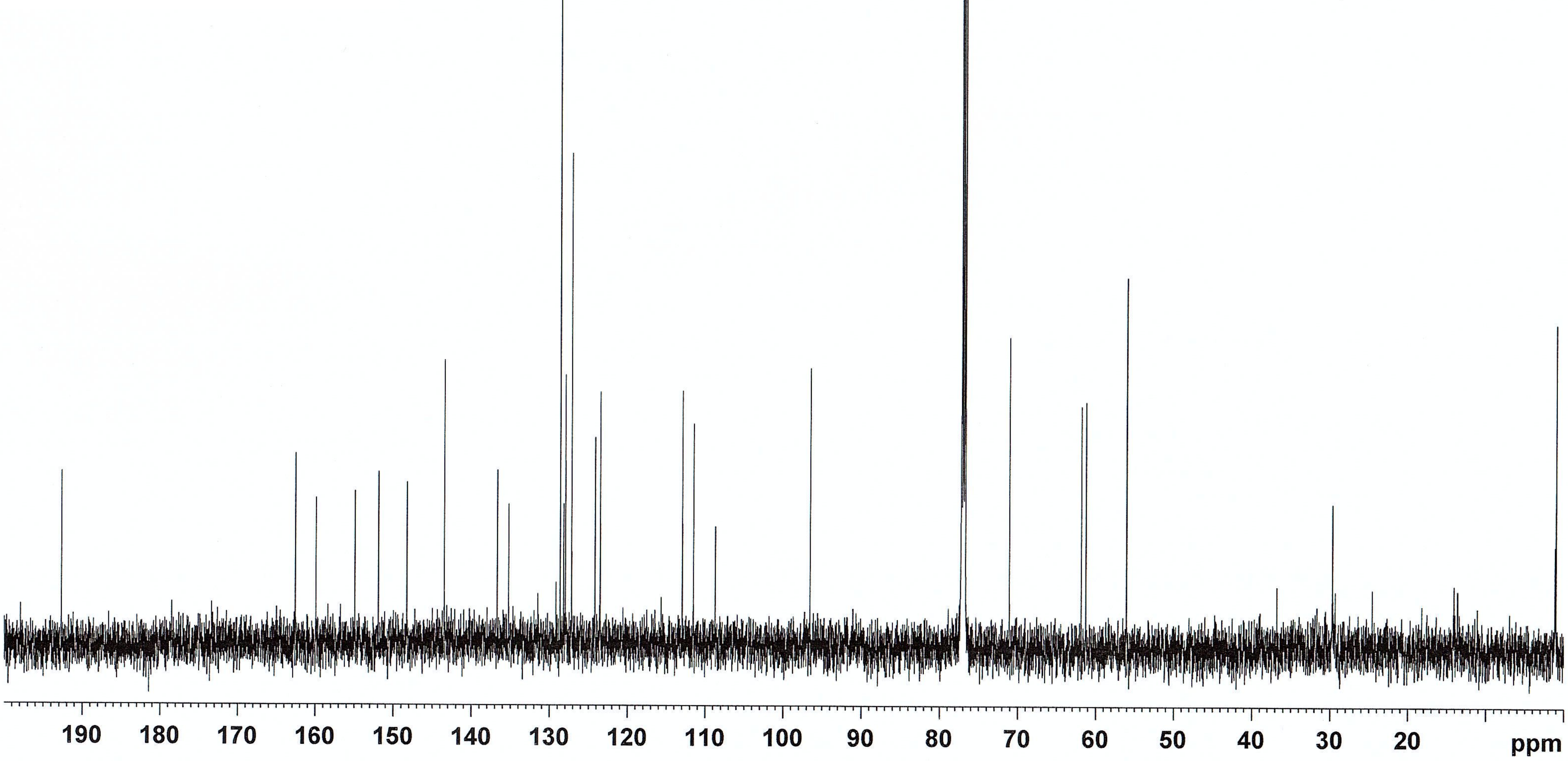

192.73
162.63
159.97
154.97
151.95
148.32
143.54
136.75
135.29
128.70
128.24
128.03
127.24
124.22
123.56
113.01
111.58
108.76
96.60
71.12
61.91
61.32
56.12
56.08
29.72
190
180
170
160
150
140
130
120
110
100
90
80
70
60
50
40
30
20
ppm

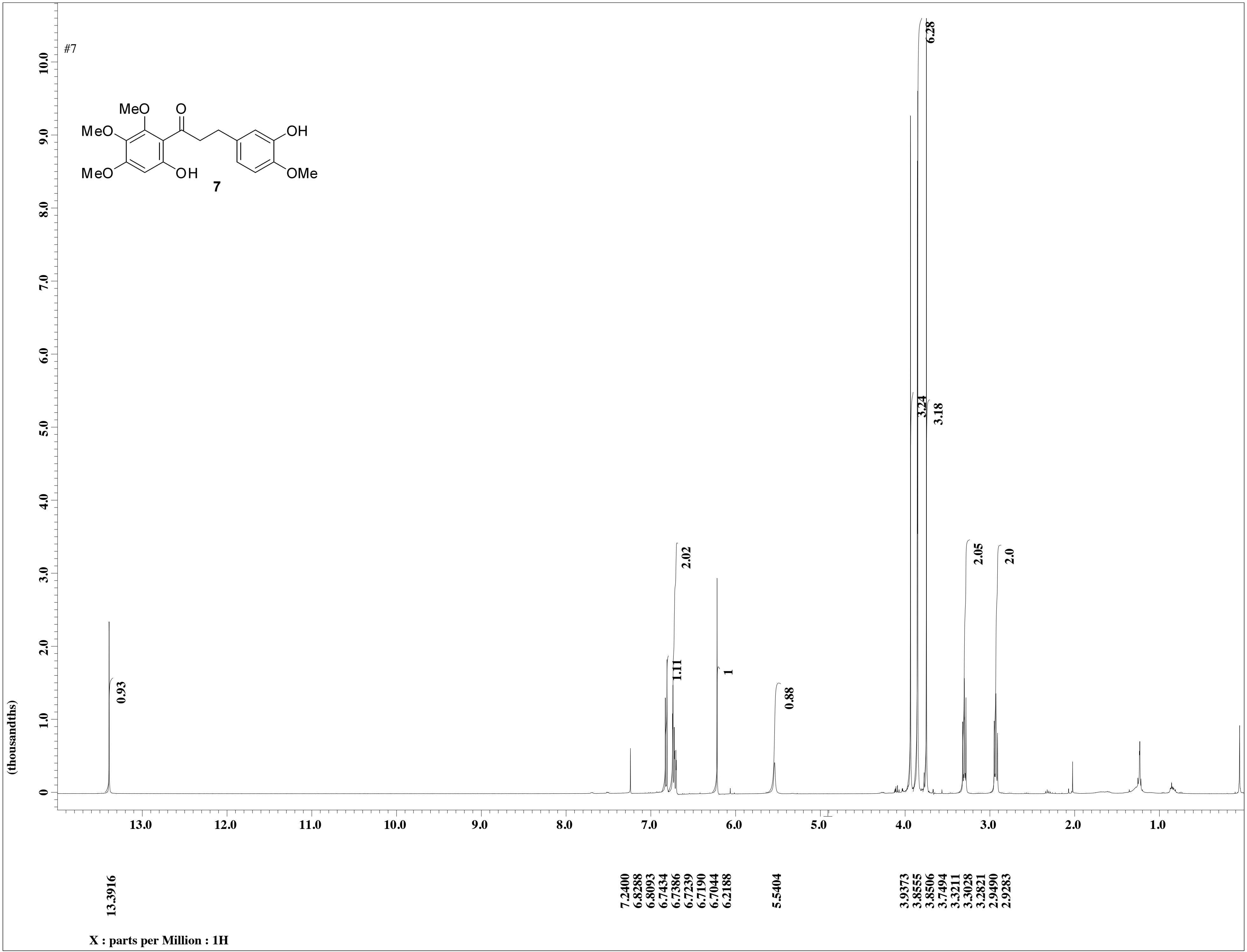
#7
MeO
O
MeO
OH
MeO
OH
7
OMe
6.28
3.24
3.18
2.05
2.0
2.02
1.11
1
0.88
0.93
(thousandths)
0
1.0
2.0
3.0
4.0
5.0
6.0
7.0
8.0
9.0
10.0
13.0
12.0
11.0
10.0
9.0
8.0
7.0
6.0
5.0
4.0
3.0
2.0
1.0
13.3916
7.2400
6.8288
6.8093
6.7434
6.7386
6.7239
6.7190
6.7044
6.2188
5.5404
3.9373
3.8555
3.8506
3.7494
3.3211
3.3028
3.2821
2.9490
2.9283
X : parts per Million : 1H

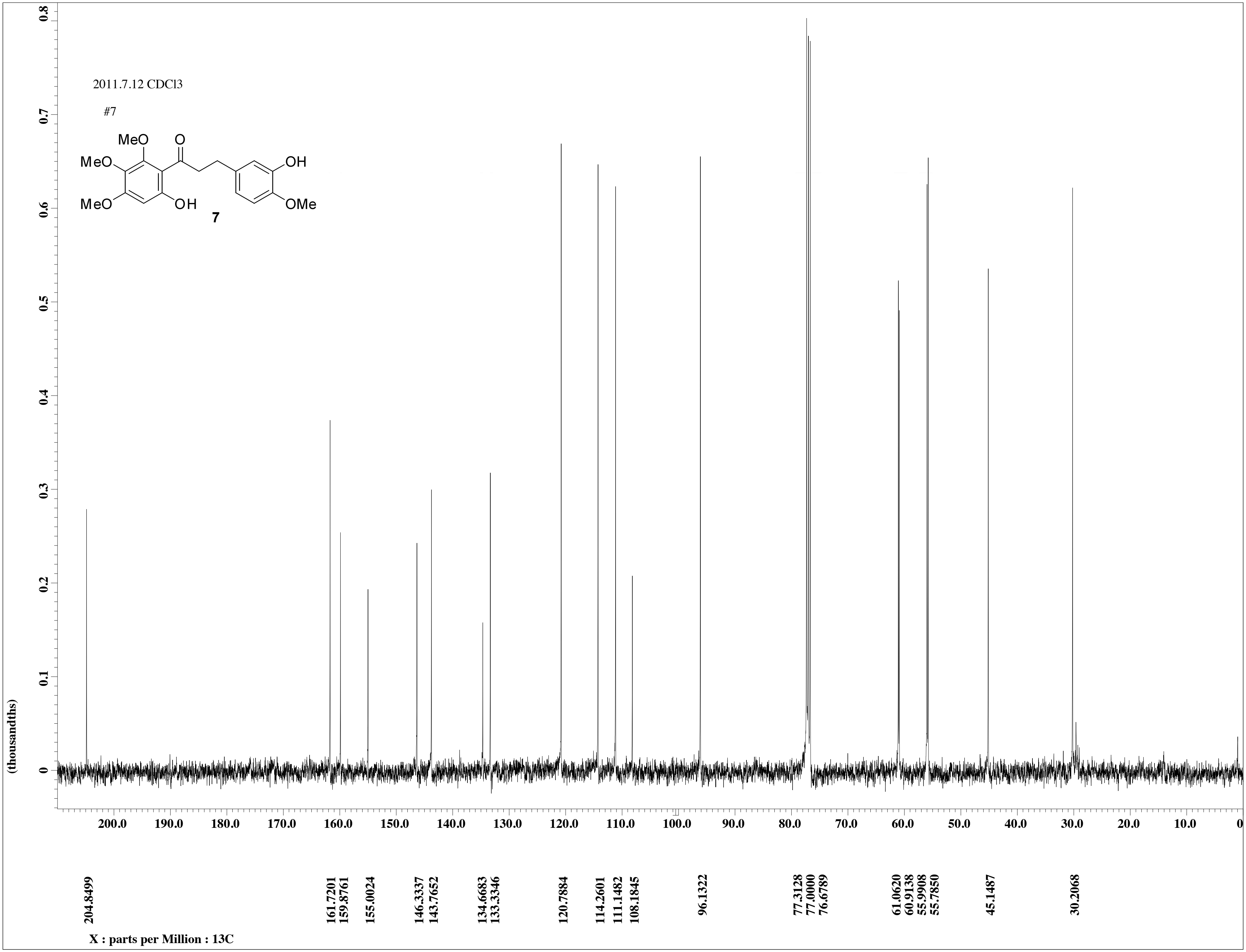
2011.7.12 CDCl3
#7
MeO
O
MeO
OH
MeO
OH
OMe
7
(thousandths)
0.8
0.7
0.6
0.5
0.4
0.3
0.2
0.1
0
200.0
190.0
180.0
170.0
160.0
150.0
140.0
130.0
120.0
110.0
100.0
90.0
80.0
70.0
60.0
50.0
40.0
30.0
20.0
10.0
0
204.8499
161.7201
159.8761
155.0024
146.3337
143.7652
134.6683
133.3346
120.7884
114.2601
111.1482
108.1845
96.1322
77.3128
77.0000
76.6789
61.0620
60.9138
55.9908
55.7850
45.1487
30.2068
X : parts per Million : 13C

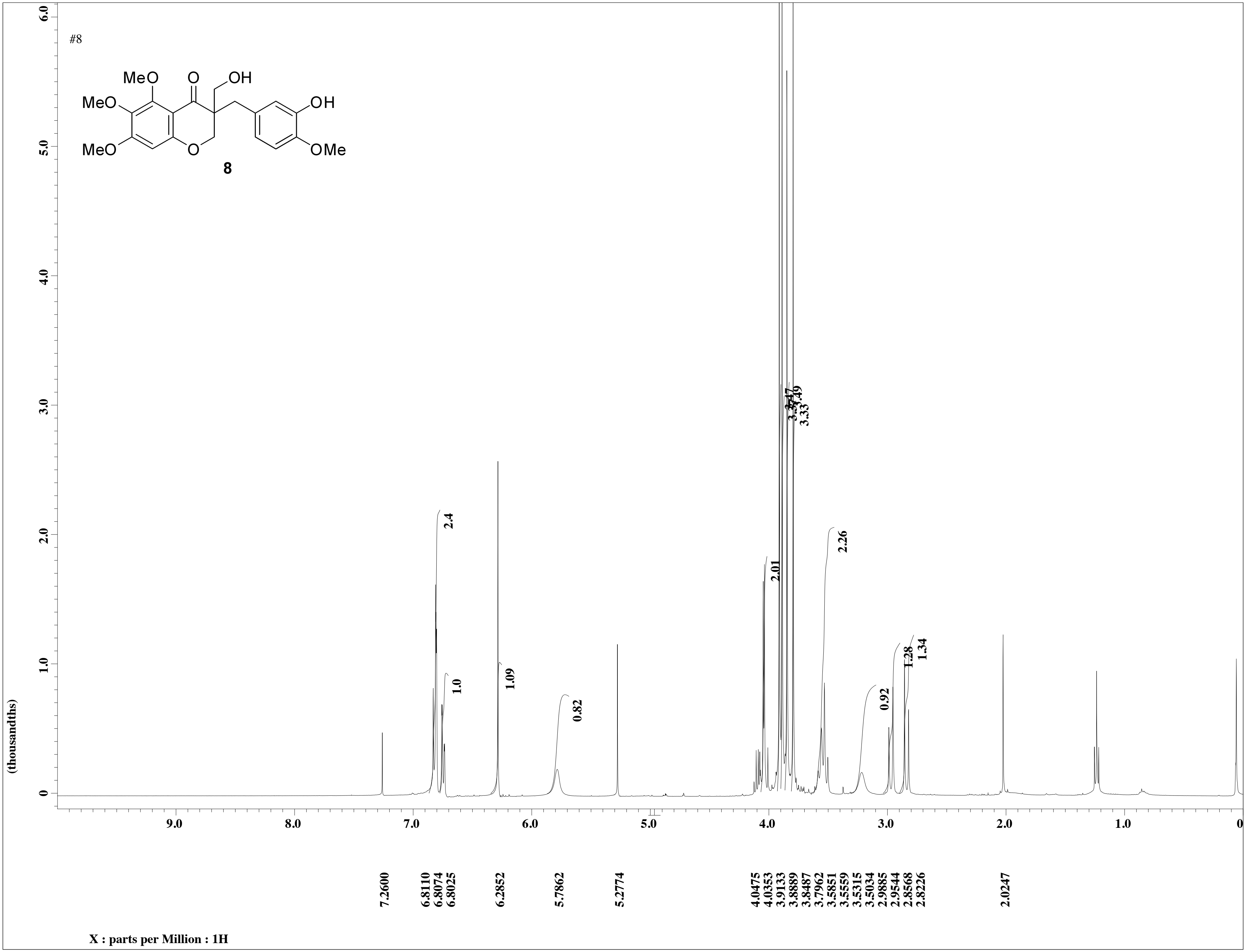
#8
MeO
O
OH
MeO
OH
MeO
O
OMe
8
(thousandths)
6.0
5.0
4.0
3.0
2.0
1.0
0
2.4
1.0
1.09
0.82
2.01
3.33
2.26
0.92
1.28
1.34
9.0 8.0 7.0 6.0 5.0 4.0 3.0 2.0 1.0 0
7.2600
6.8110
6.8074
6.8025
6.2852
5.7862
5.2774
4.0475
4.0353
3.9133
3.8889
3.8487
3.7962
3.5851
3.5559
3.5315
3.5034
2.9885
2.9544
2.8568
2.8226
2.0247
X : parts per Million : 1H

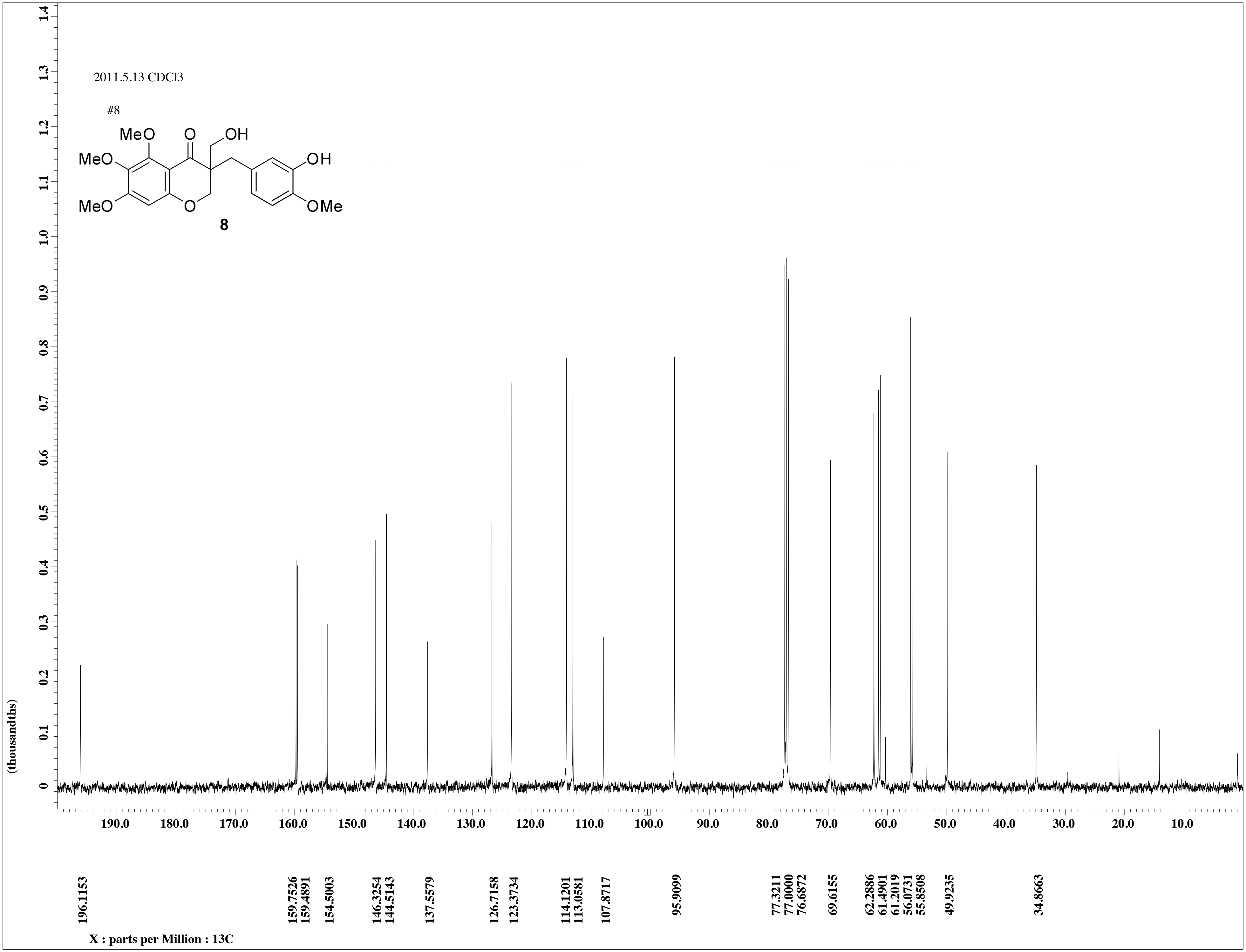
2011.5.13 CDCl3
#8
MeO
O
OH
MeO
OH
MeO
O
OMe
8
(thousandths)
1.4
1.3
1.2
1.1
1.0
0.9
0.8
0.7
0.6
0.5
0.4
0.3
0.2
0.1
0
190.0
180.0
170.0
160.0
150.0
140.0
130.0
120.0
110.0
100.0
90.0
80.0
70.0
60.0
50.0
40.0
30.0
20.0
10.0
196.1153
159.7526
159.4891
154.5003
146.3254
144.5143
137.5579
126.7158
123.3734
114.1201
113.0581
107.8717
95.9099
77.3211
77.0000
76.6872
69.6155
62.2886
61.4901
61.2019
56.0731
55.8508
49.9235
34.8663
X : parts per Million : 13C

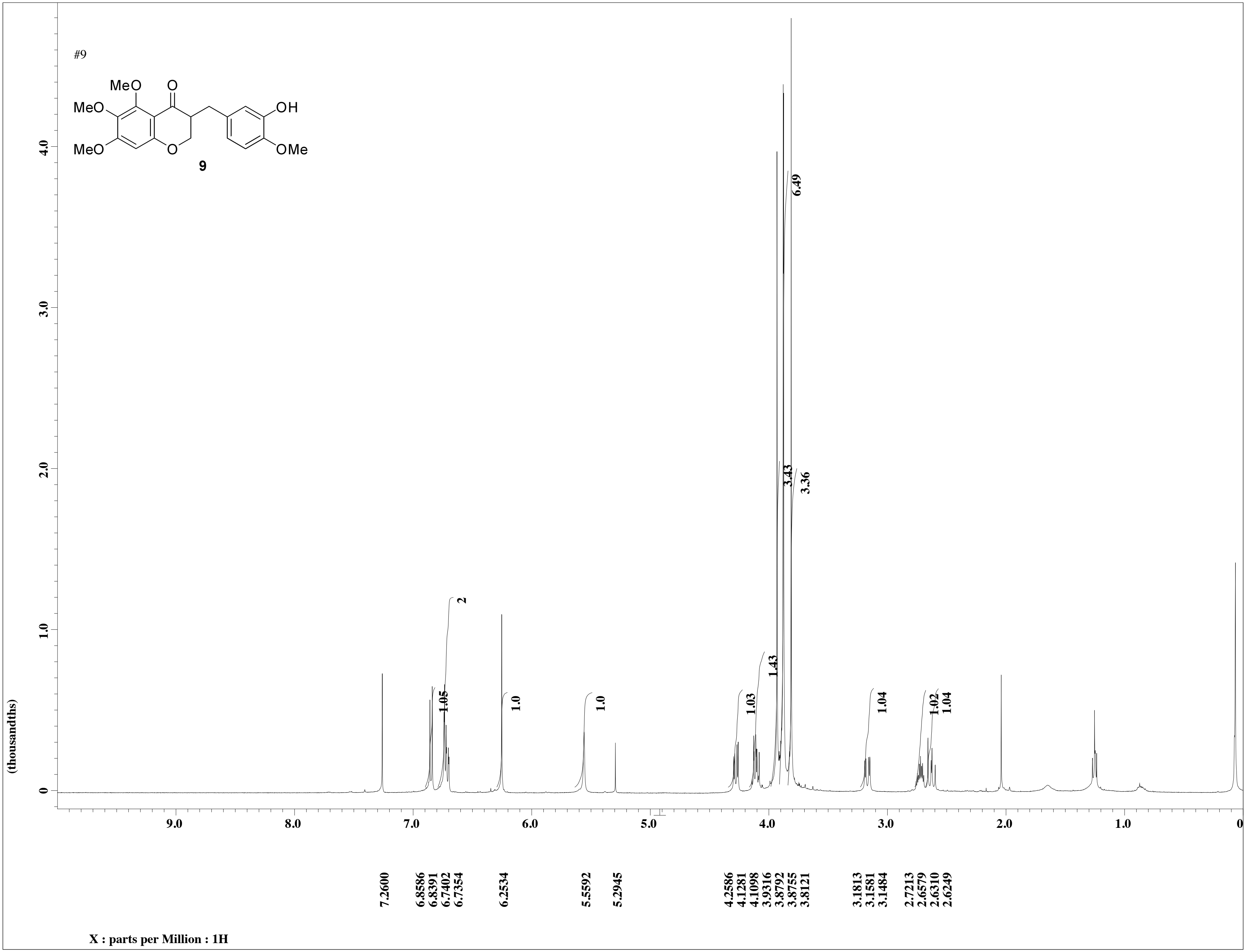
#9
MeO
MeO
MeO
O
OH
OMe
O
9
(thousandths)
X : parts per Million : 1H
7.2600
6.8586
6.8391
6.7402
6.7354
6.2534
5.5592
5.2945
4.2586
4.1281
4.1098
3.9316
3.8792
3.8755
3.8121
3.1813
3.1581
3.1484
2.7213
2.6579
2.6310
2.6249
2
1.05
1.0
1.0
1.03
1.43
3.43
6.49
3.36
1.04
1.02
1.04

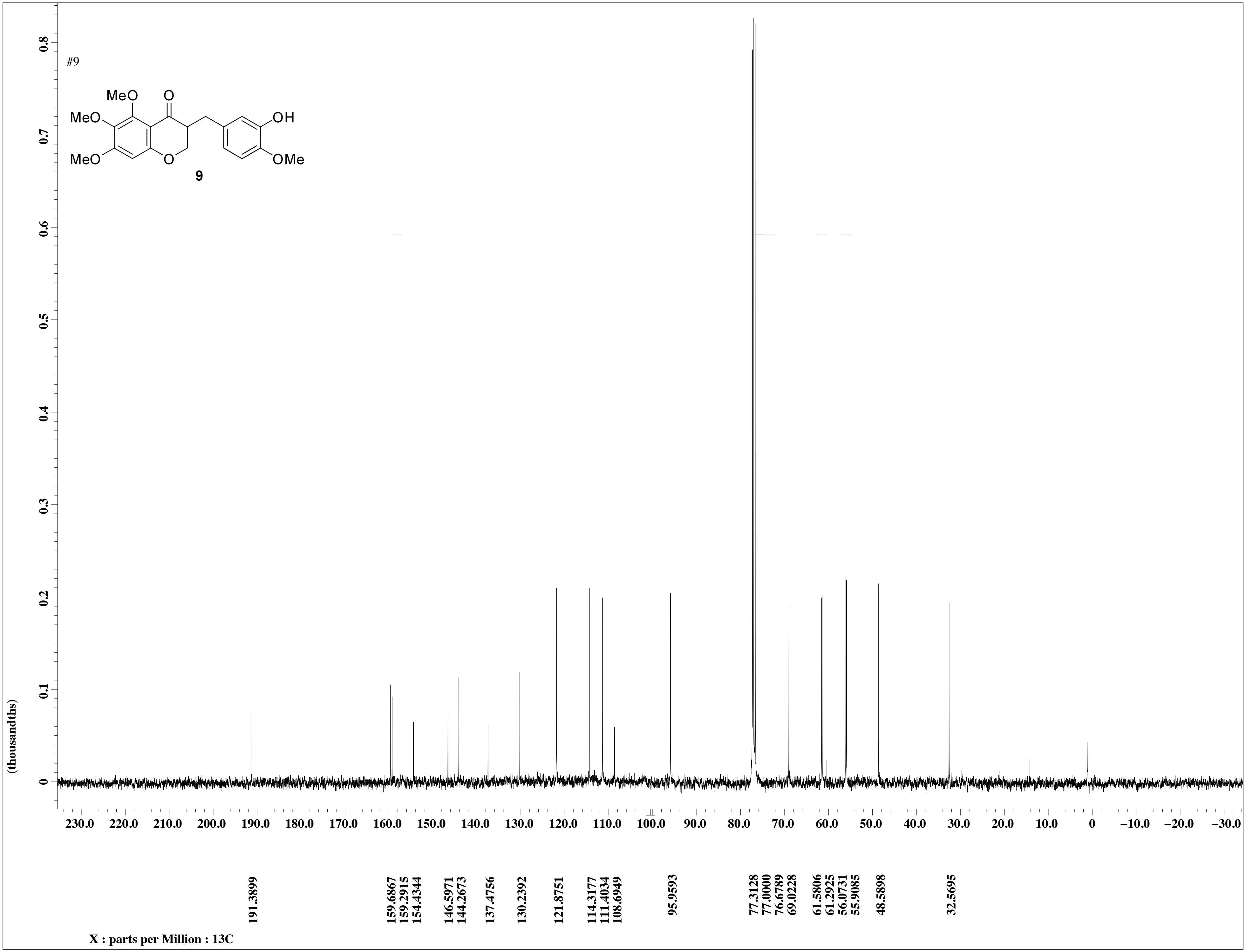
#9
MeO
O
MeO
OH
MeO
O
OMe
9
(thousandths)
0.8
0.7
0.6
0.5
0.4
0.3
0.2
0.1
0
230.0 220.0 210.0 200.0 190.0 180.0 170.0 160.0 150.0 140.0 130.0 120.0 110.0 100.0 90.0 80.0 70.0 60.0 50.0 40.0 30.0 20.0 10.0 0 −10.0 −20.0 −30.0
191.3899
159.6867
159.2915
154.4344
146.5971
144.2673
137.4756
130.2392
121.8751
114.3177
111.4034
108.6949
95.9593
77.3128
77.0000
76.6789
69.0228
61.5806
61.2925
56.0731
55.9085
48.5898
32.5695
X : parts per Million : 13C

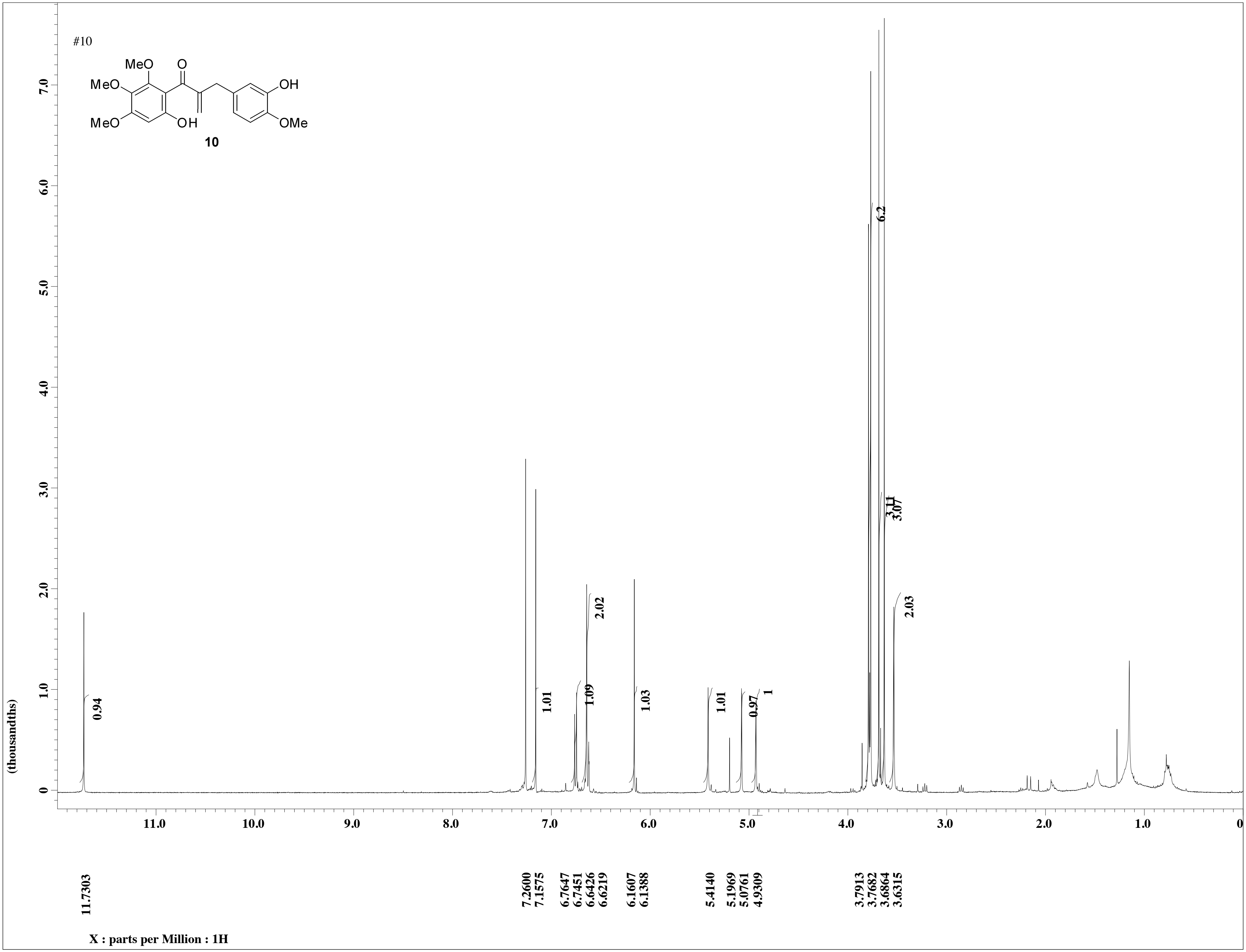

#10
MeO
O
MeO
OH
MeO
OH
OMe
10
7.0
6.0
5.0
4.0
3.0
2.0
1.0
0
(thousandths)
0.94
1.01
1.09
2.02
1.03
1.01
0.97
1
6.2
3.11
3.07
2.03
11.0
10.0
9.0
8.0
7.0
6.0
5.0
4.0
3.0
2.0
1.0
0
11.7303
7.2600
7.1575
6.7647
6.7451
6.6426
6.6219
6.1607
6.1388
5.4140
5.1969
5.0761
4.9309
3.7913
3.7682
3.6864
3.6315
X : parts per Million : 1H

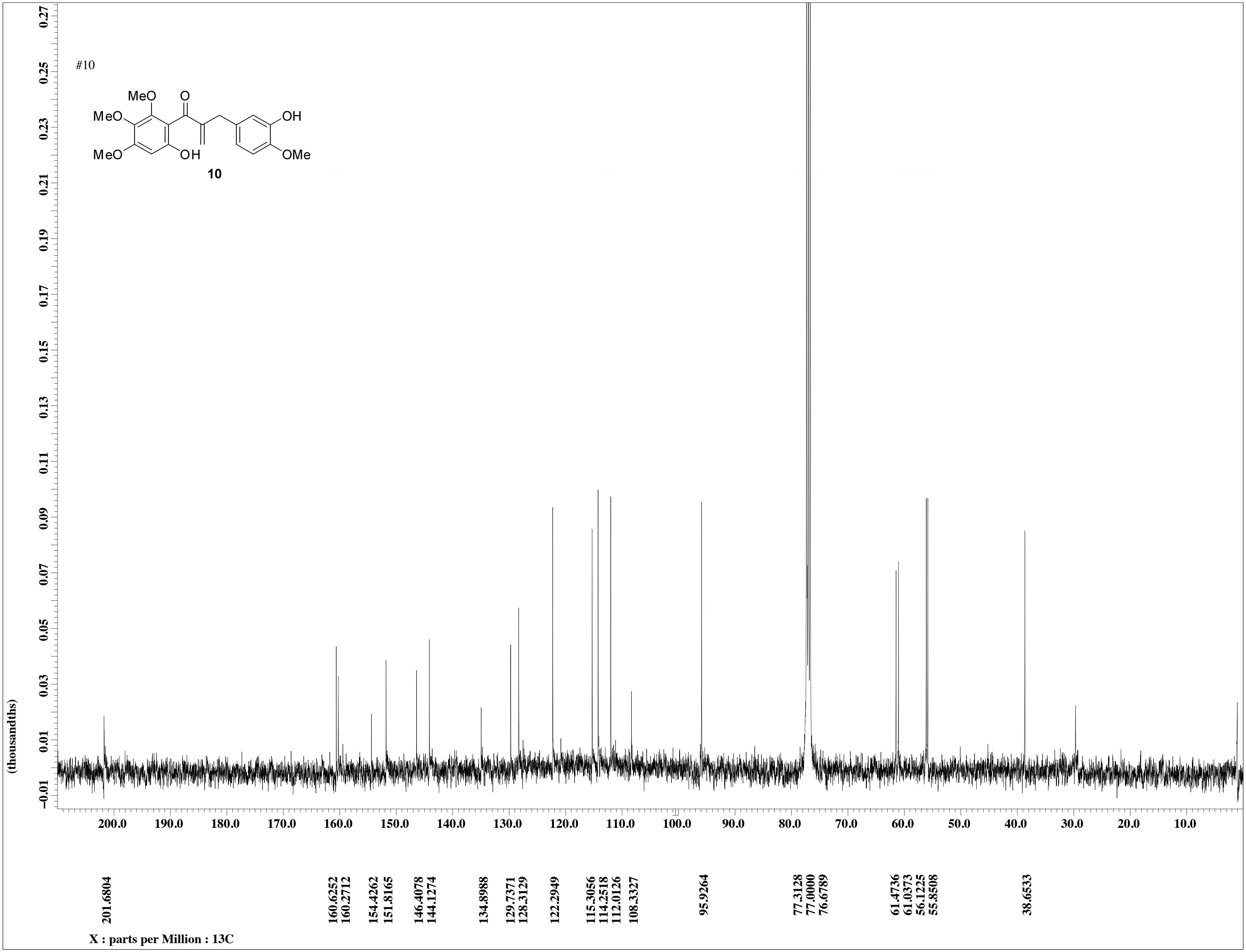
#10
MeO
O
MeO
OH
MeO
OH
OMe
10
(thousandths)
0.27
0.25
0.23
0.21
0.19
0.17
0.15
0.13
0.11
0.09
0.07
0.05
0.03
0.01
−0.01
200.0
190.0
180.0
170.0
160.0
150.0
140.0
130.0
120.0
110.0
100.0
90.0
80.0
70.0
60.0
50.0
40.0
30.0
20.0
10.0
201.6804
160.6252
160.2712
154.4262
151.8165
146.4078
144.1274
134.8988
129.7371
128.3129
122.2949
115.3056
114.2518
112.0126
108.3327
95.9264
77.3128
77.0000
76.6789
61.4736
61.0373
56.1225
55.8508
38.6533
X : parts per Million : 13C

SH-11052

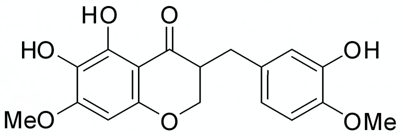

2

Methanol-d4, 150MHz

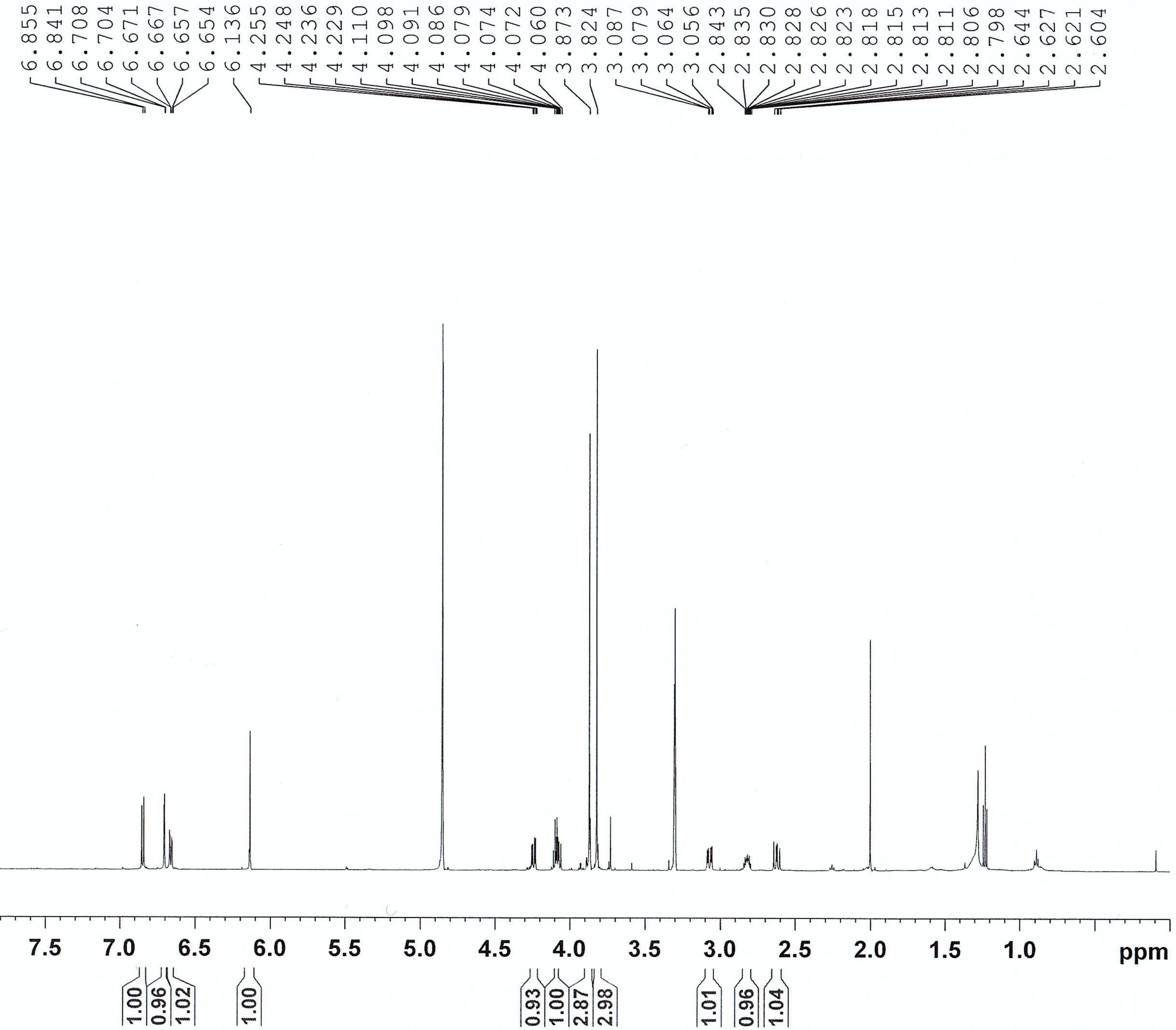

Carbon SH-11052

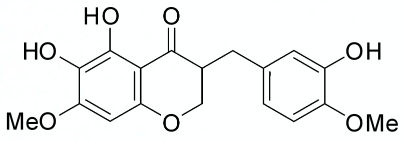

2

Methanol-d4, 150MHz

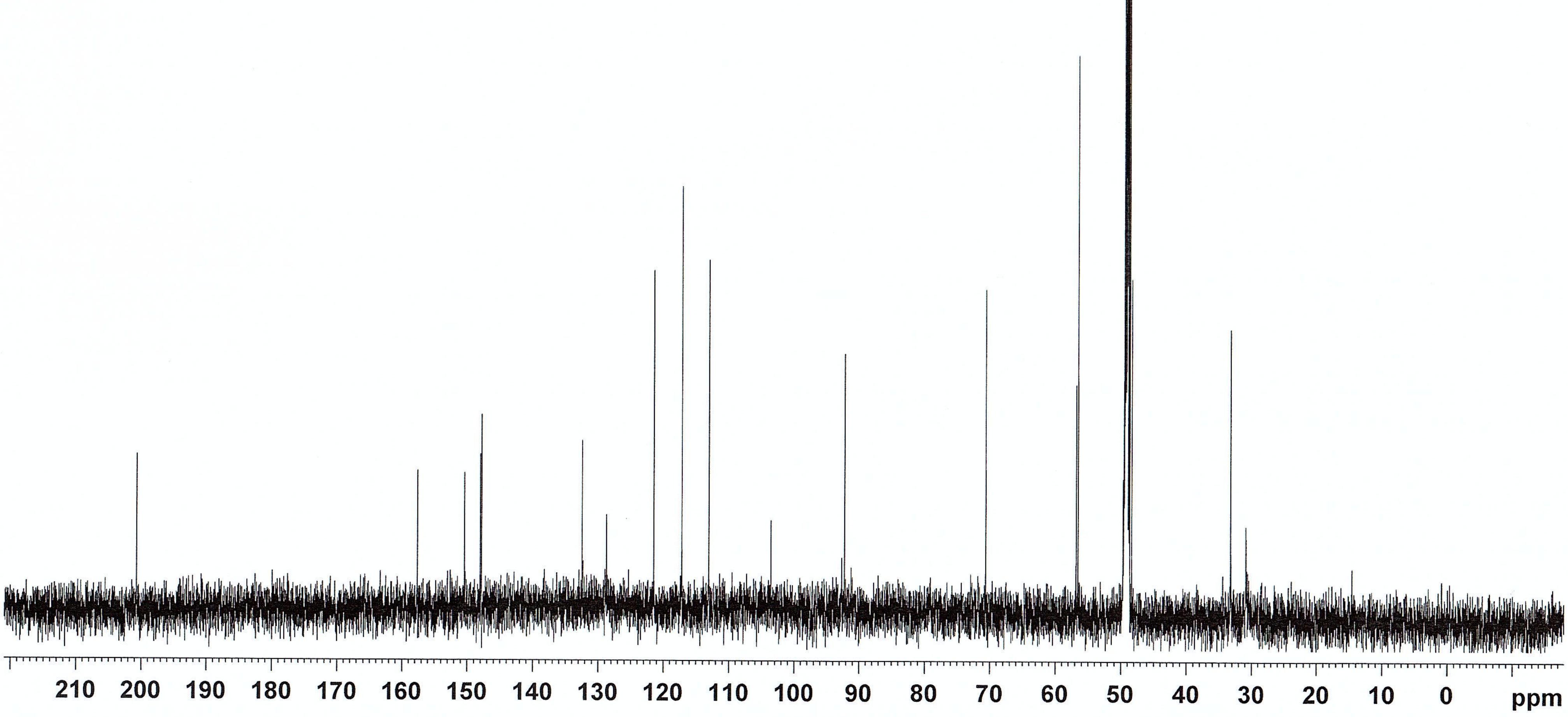

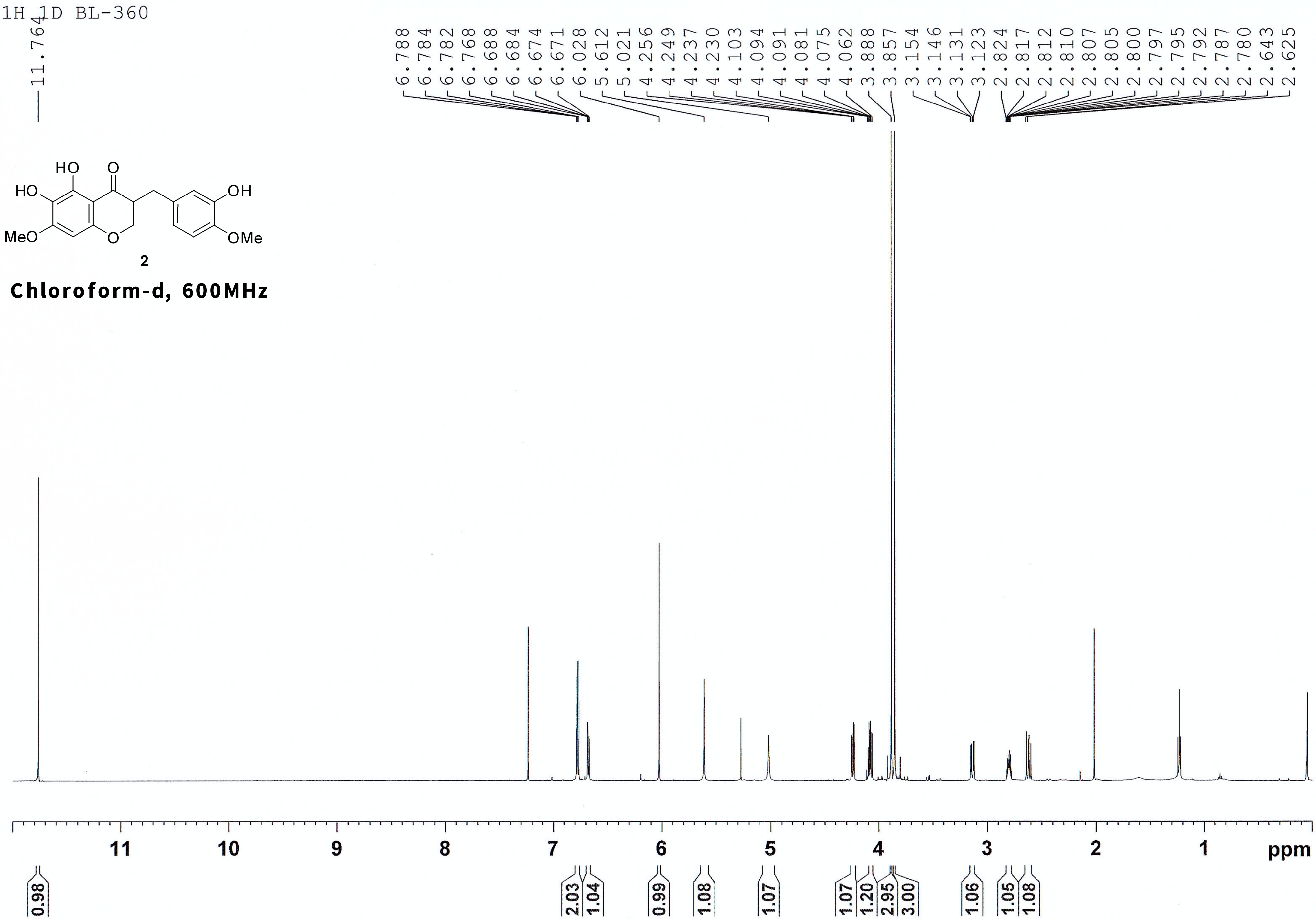
1H 1D BL-360
11.764
6.788
6.784
6.782
6.768
6.688
6.684
6.674
6.671
6.028
5.612
5.021
4.256
4.249
4.237
4.230
4.103
4.094
4.091
4.081
4.075
4.062
3.888
3.857
3.154
3.146
3.131
3.123
2.824
2.817
2.812
2.810
2.807
2.805
2.800
2.797
2.795
2.792
2.787
2.780
2.643
2.625
HO
O
HO
OH
MeO
O
OMe
2
Chloroform-d, 600MHz
11
10
9
8
7
6
5
4
3
2
1
ppm
0.98
2.03
1.04
0.99
1.08
1.07
1.07
1.20
2.95
3.00
1.06
1.05
1.08

Carbon 1DBL-360

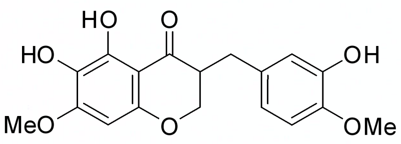

2

Chloroform-d, 150MHz

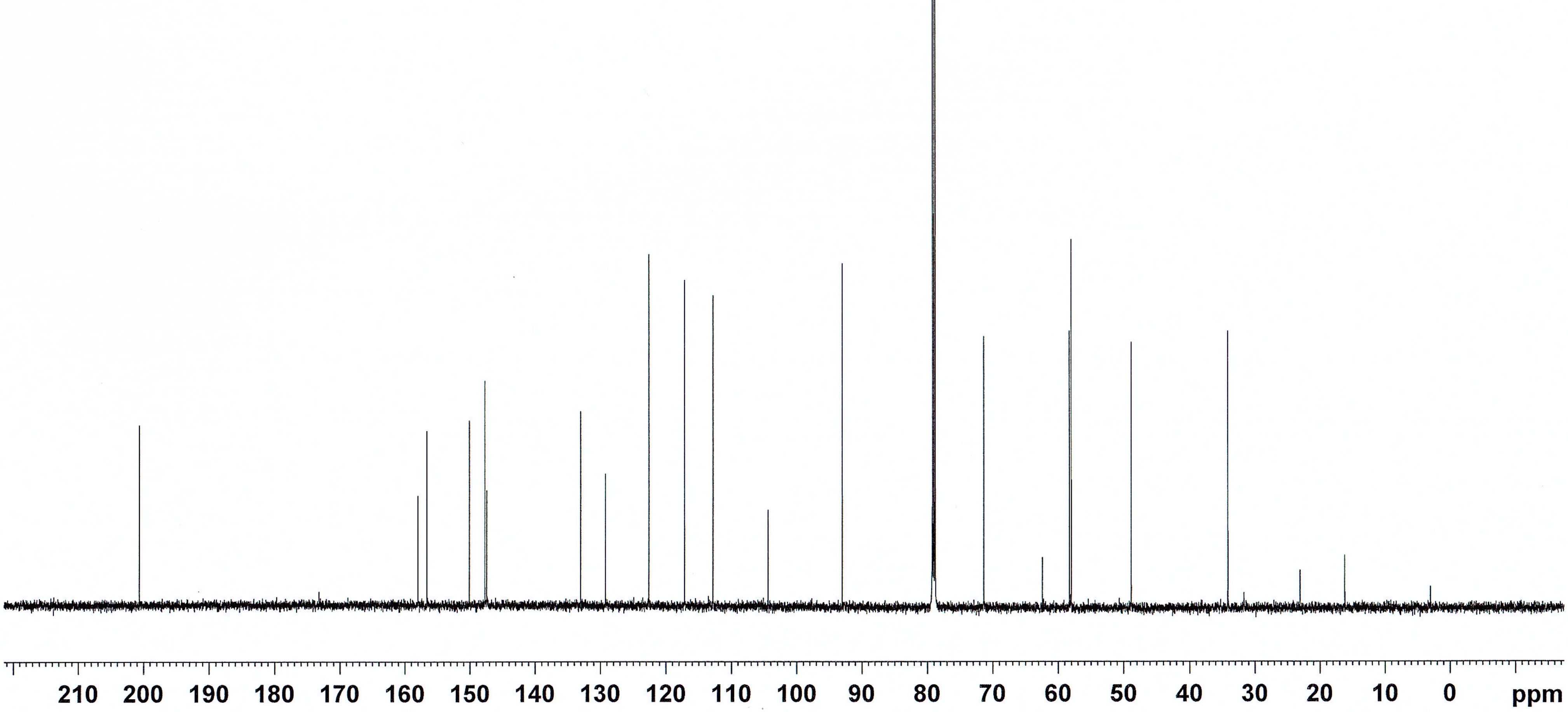

H noesy

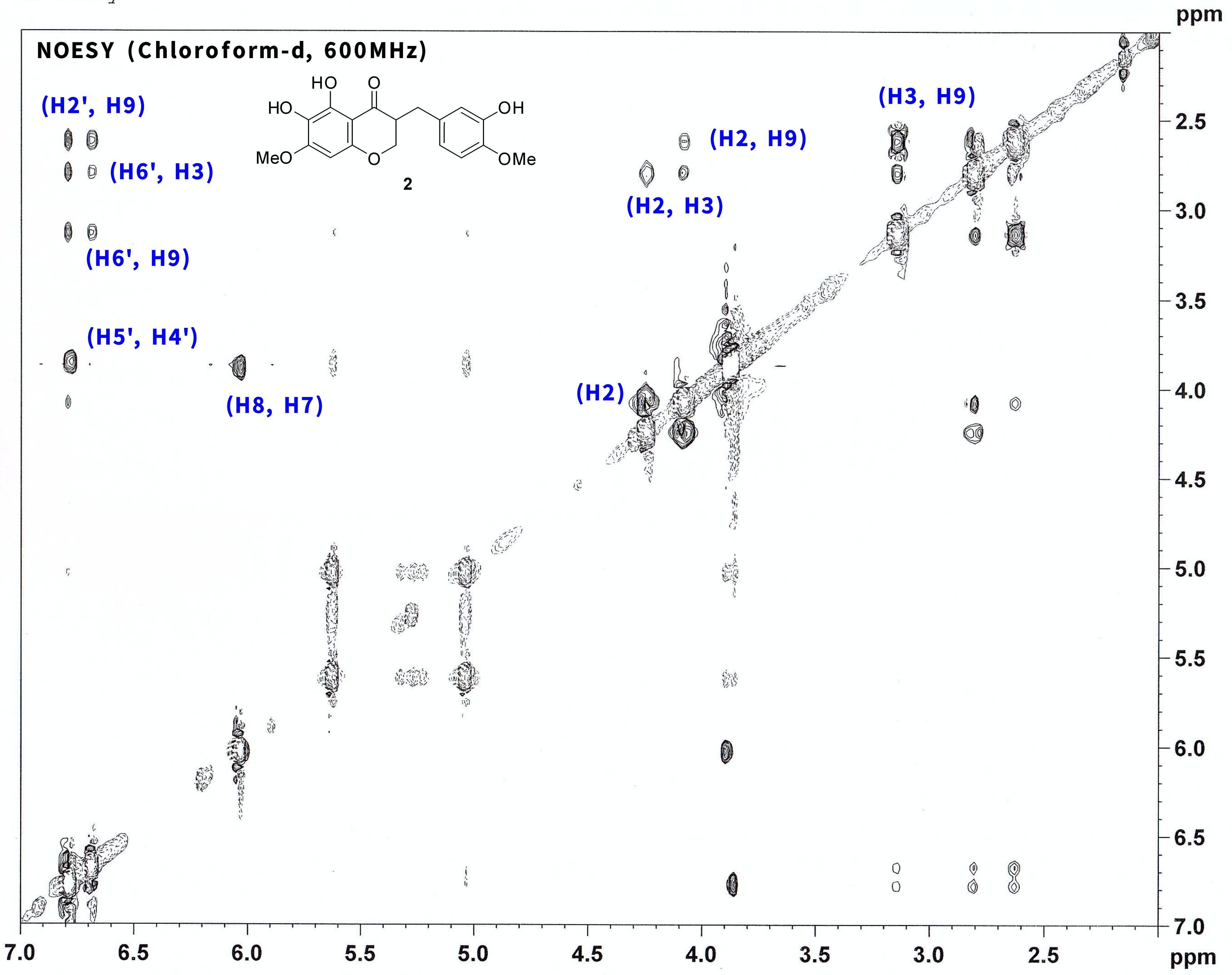

Current Data Parameters
NAME BL
EXPNO 604
PROCNO 1

F2 - Acquisition Parameters
Date_ 20140102
Time 14.17
INSTRUM spect
PROBHD 5 mm CPTCI 1H-
PULPROG noesygpphpp
TD 2048
SOLVENT CDCl3
NS 48
DS 32
SWH 12019.230 Hz
FIDRES 5.868765 Hz
AQ 0.0851968 sec
RG 35.72
DW 41.600 usec
DE 10.00 usec
TE 298.0 K
D0 0.00003154 sec
D1 2.00000000 sec
D8 0.50000000 sec
D11 0.03000000 sec
D12 0.00002000 sec
D16 0.00020000 sec
IN0 0.00008320 sec

======== CHANNEL f1 ========
SFO1 600.2436014 MHz
NUC1 1H
P1 7.90 usec
P2 15.80 usec
P17 2500.00 usec
PLW1 7.00000000 W
PLW10 0.64626002 W

====== GRADIENT CHANNEL =====
GPNAM[1] SMSQ10.100
GPZ1 40.00 %
P16 1000.00 usec

F1 - Acquisition parameters
TD 512
SFO1 600.2436 MHz
FIDRES 23.475060 Hz
SW 20.024 ppm
FnMODE States-TPPI

F2 - Processing parameters
SI 1024
SF 600.2400233 MHz
WDW QSINE
SSB 2
LB 0 Hz
GB 0
PC 1.40

F1 - Processing parameters
SI 1024
MC2 States-TPPI
SF 600.2400284 MHz
WDW QSINE
SSB 2
LB 0 Hz
GB 0

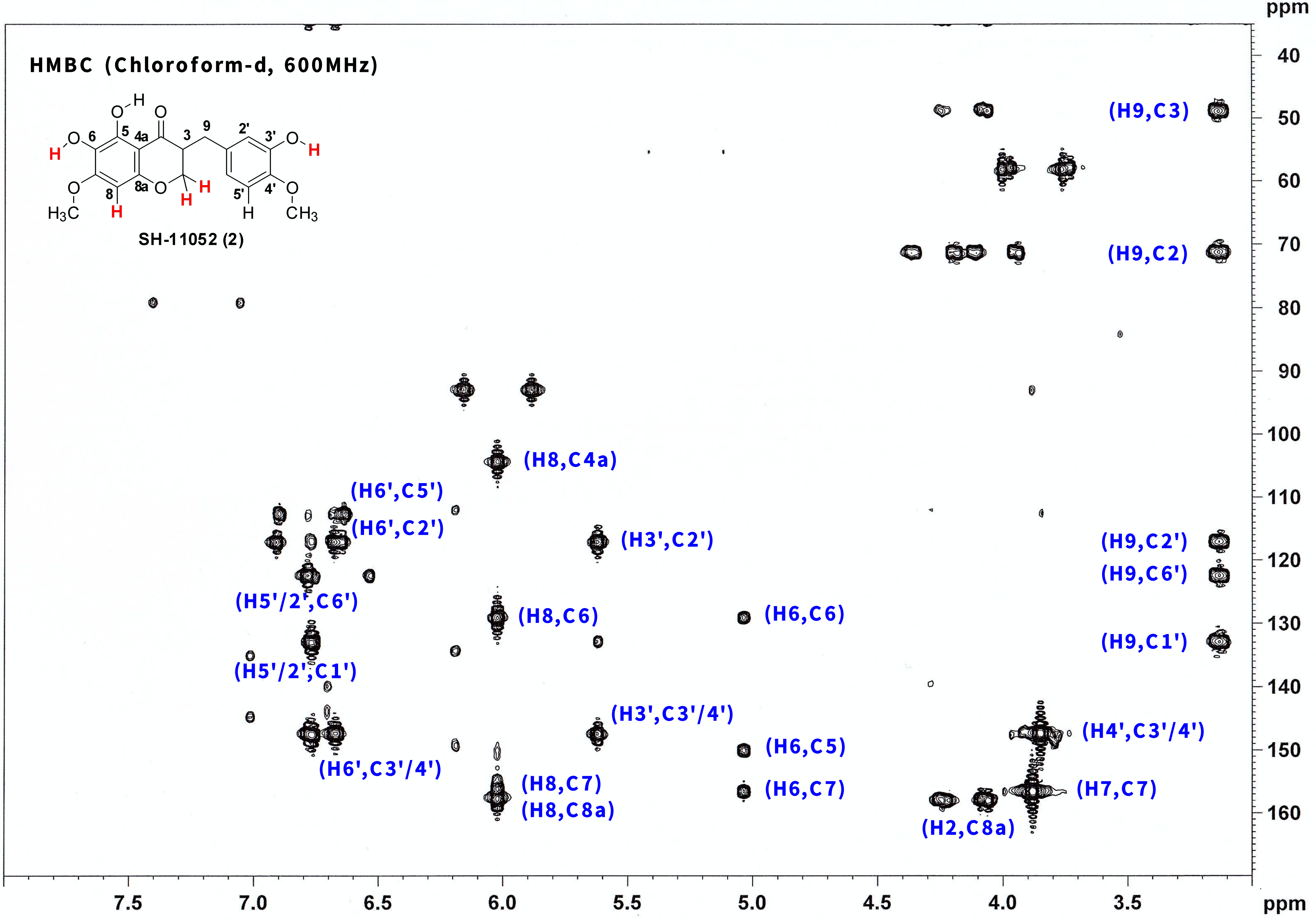

HMBC (Chloroform-d, 600MHz)
SH-11052 (2)
(H9,C3)
(H9,C2)
(H8,C4a)
(H6',C5')
(H6',C2')
(H3',C2')
(H9,C2')
(H9,C6')
(H5'/2',C6')
(H8,C6)
(H6,C6)
(H9,C1')
(H5'/2',C1')
(H3',C3'/4')
(H4',C3'/4')
(H6,C5)
(H6',C3'/4')
(H8,C7)
(H6,C7)
(H7,C7)
(H8,C8a)
(H2,C8a)
ppm
ppm

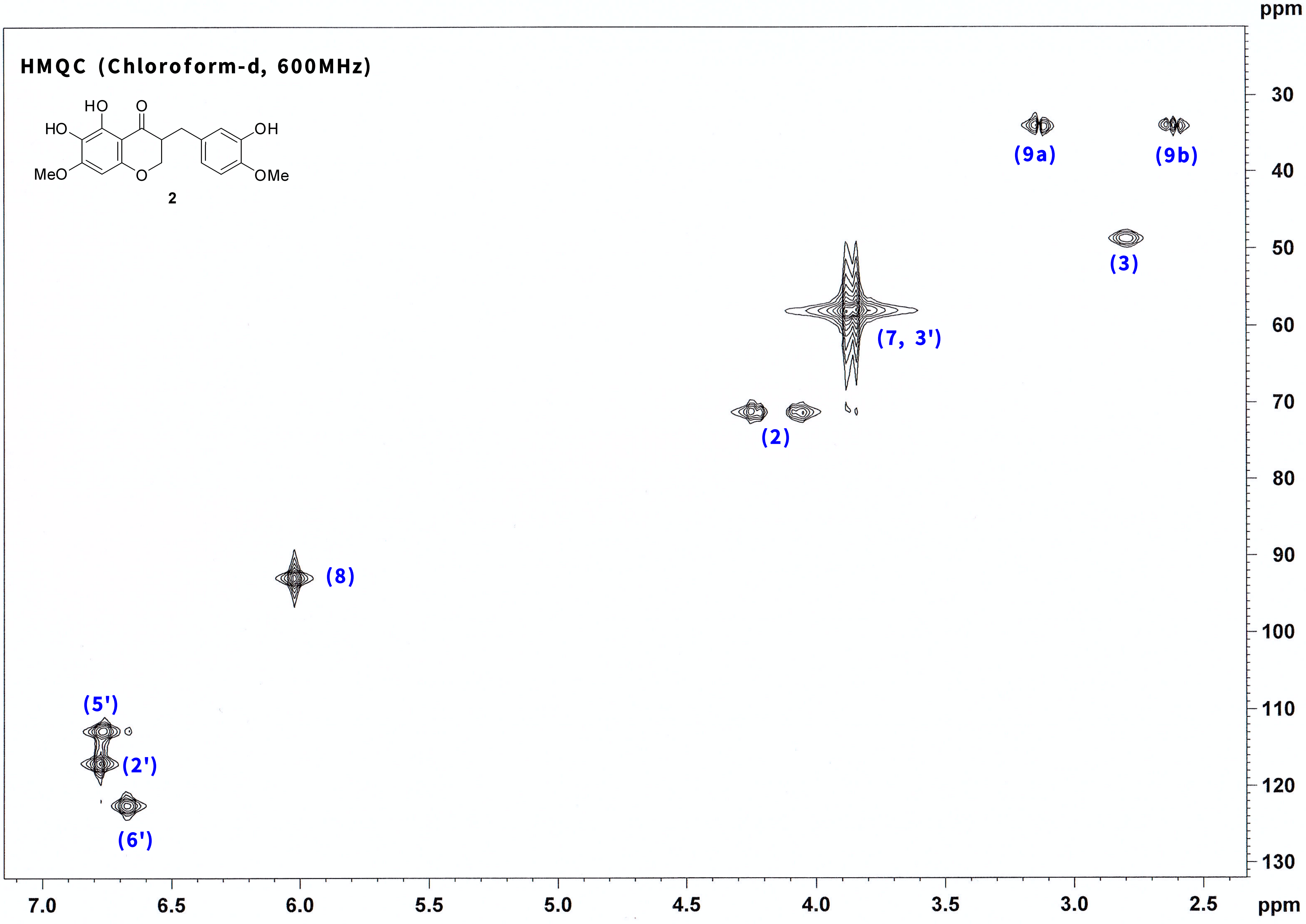

HMQC (Chloroform-d, 600MHz)
HO
O
HO
OH
MeO
O
OMe
2
(9a)
(9b)
(3)
(7, 3')
(2)
(8)
(5')
(2')
(6')
ppm
30
40
50
60
70
80
90
100
110
120
130
7.0
6.5
6.0
5.5
5.0
4.5
4.0
3.5
3.0
2.5
ppm
